# Incidence, Risk and Trends of Multiple Primary Cancers in Patients With Colorectal Cancer: Evidence From the South Australian Cancer Registry

**DOI:** 10.1002/cam4.70984

**Published:** 2025-05-30

**Authors:** Mulugeta Melku, Oliver G. Best, Jean M. Winter, Lauren A. Thurgood, Muktar Ahmed, Ganessan Kichenadasse, Murthy Mittinty, Molla M. Wassie, Erin L. Symonds

**Affiliations:** ^1^ Flinders Health and Medical Research Institute, College of Medicine and Public Health Flinders University Adelaide South Australia Australia; ^2^ Department of Hematology and Immunohematology, School of Biomedical and Laboratory Sciences, College of Medicine and Health Science University of Gondar Gondar Ethiopia; ^3^ Medical Oncology Department, Flinders Centre for Innovation in Cancer Flinders Medical Centre, South Adelaide Local Health Network Adelaide South Australia Australia; ^4^ Gastroenterology Department Flinders Medical Centre, South Adelaide Local Health Network Adelaide South Australia Australia

**Keywords:** colorectal cancer survivors, incidence, multiple primary cancer, risk, trend

## Abstract

**Background:**

Colorectal cancer (CRC) is the fourth most diagnosed cancer in Australia. With advancements in treatment and an increase in survival rates, CRC survivors face an elevated risk of developing multiple primary cancers (MPCs), presenting a clinical challenge. Therefore, this study aimed to estimate the incidence, trend and risk of MPCs after a diagnosis of CRC in the South Australian population.

**Methods:**

This study analysed South Australian Cancer Registry data on individuals diagnosed with CRC as their first cancer from 1982 to 2017. The incidence of MPCs was assessed using cumulative incidence functions, and age‐standardised rates were estimated. Poisson regression was used to determine the risk, and standardised incidence ratios (SIR) and absolute excess risks (AER) were estimated. Trends over time were analysed using Joinpoint regression.

**Results:**

The study included 26,729 CRC survivors. Of the cohort, 15% (3917) developed 4453 MPCs, with 96% diagnosed six or more months after index CRC. The cumulative incidence of MPCs was 22.5% (95% CI: 21.6–23.4). The median follow‐up time until MPC diagnosis was 6.4 years. Common MPCs included prostate (18.9%), subsequent CRC (13.1%), lung (10.8%), haematological (10.2%) and breast (8.0%) cancers. The overall risk of MPCs was higher in CRC survivors (SIR: 1.12, 95% CI: 1.09–1.16; AER: 22.6 per 10,000) compared to the incidence in the general South Australian population. The incidence of MPCs has increased over time (annual percentage change = 1.95, 95% CI: 1.33–2.51).

**Conclusions:**

CRC survivors are at increased risk of subsequent cancers, highlighting the need for targeted surveillance, particularly for prostate, lung, breast and blood cancers, for early detection and treatment.

## Graphical abstract

In this study, the risk and trends of multiple primary cancers were estimated using large state‐wide data collected over 35 years. The findings revealed a significantly higher risk of subsequent primary cancers, with an increasing trend over time. These results underscore the need for informed strategies to enhance and refine surveillance programmes for the early detection of subsequent cancers. Such measures could improve not only overall survival but also the quality of survivorship by addressing long‐term health risks more effectively.

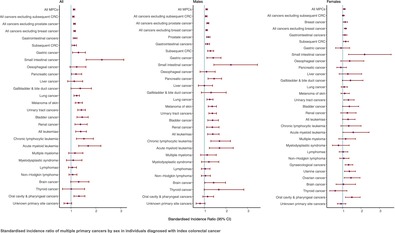

**Abbreviations**
AER: absolute excess risk
APC: annual percentage changes
ASR: age‐standardised rate
CRC: colorectal cancer
GI: gastrointestinal
IACR: International Association of Cancer Registries
IARC: International Agency for research on cancer
ICD‐O‐3: International Classification of Diseases for Oncology third edition
MPC: multiple primary cancer
SACR: South Australian Cancer Registry
SIR: standardised incidence ratio

## 1 Introduction

Colorectal cancer (CRC) is the third most common cancer diagnosed globally, following breast and lung cancer, and the fourth most common based on the age‐standardised rate (ASR), following breast, prostate and lung cancers [1]. It is projected that between 2022 and 2040, there will be a 47.7% increase in the incidence of CRC cases globally [2]. In Australia, the number of new CRC cases increased from 6991 in 1982 to 14,534 in 2020, making it the fourth most commonly diagnosed cancer and the second‐highest cause of cancer‐related death [3].

Over the past four decades, there have been significant advances in screening for early detection of cancer, improved diagnostic tools, more targeted treatment options and improved awareness in the community about cancer and its prevention [4]. These have contributed to a decline in the mortality rate and an improved overall survival rate [5]. The Global Burden of Disease study reported that the age‐standardised cancer mortality rate declined by 5.9%, with a 0.7% annual rate of change from 2010 to 2019 globally [6]. A decrease in age‐standardised mortality, along with an increasing trend in the five‐year survival rates among CRC patients, has also been observed [7]. In Australia, there has been a significant improvement in the five‐year relative survival over time, with the rate increasing from 54.9% in 1991–1995 to 71.3% in 2016–2020.

While patient outcomes have improved, CRC survivors face an increased risk of developing one or more cancers in another part of the colon, rectum or other primary sites, herein collectively referred to as multiple primary cancers (MPCs) [8, 9]. MPCs are defined as the occurrence of more than one cancer that arises in an individual, either as a synchronous or metachronous primary cancer. MPCs can occur in the same or different primary site or tissue and usually harbour distinct histological features; they are not an extension, recurrence or metastasis of the original cancer [10]. Population‐based cancer registry studies report an increasing incidence of MPCs among CRC survivors, particularly in developed countries. For instance, 11.5% and 7.6% of CRC patients in the United States and Italy, respectively, develop MPC six months post‐diagnosis [8, 11], while 4.3% of CRC patients in Taiwan develop MPC one year after their initial CRC diagnosis [12]. A Dutch study further reported a growing cumulative incidence of metachronous CRC with longer survival times of patients diagnosed with a primary CRC [13].

In Australia, while some evidence exists regarding the risks of MPC [14, 15, 16], it is essential to assess the current status and trend of MPC among CRC survivors, given the evolving practices in CRC management and the improved survival rates. A study from the Queensland Cancer Registry (1996–2007) found that nearly 10% of CRC survivors aged 20–79 developed MPC [14]. Another study from the New South Wales Cancer Registry revealed that 5% of survivors developed subsequent MPCs [16], while a retrospective study from the same data source (1987–1996) showed that 2.1% of CRC cases were diagnosed with a second primary CRC [15]. In addition to estimating the magnitude of MPC diagnoses, it is important to assess the cancer‐specific risk of MPC beyond the expected cancer incidence in the general population.

Evidence has shown that individuals initially diagnosed with CRC have an increased risk of developing subsequent MPCs, with a standardised incidence ratio (SIR) ranging from 1.05 to 1.73 [12, 14, 15], although other studies have disputed this [17, 18]. A study from the Taiwan Cancer Registry reported the risk of a subsequent primary cancer was not significantly elevated compared with that of the general population [18]. Furthermore, a study from South Korea revealed that the risk of subsequent primary cancer among 5‐year survivors of CRC is notably lower than the risk of cancer in the general population [17]. The risk varies according to the site/tissue, and the common types of cancers include prostate, breast, small intestine, gastric, renal, lung and haematological cancers [11, 14].

While there have been previous studies concerning the incidence and risk of MPCs, there are limited publications using more recent data, especially from Australia. Therefore, this study aimed to determine the incidence, trends and types of MPCs in CRC survivors, and to estimate the risk of MPCs in CRC survivors compared to the risk of cancer in the general population, using large‐scale, state‐wide, population‐based cancer registry data in South Australia.

## 2 Methods

### 2.1 Study Design, Setting and Population

This study was a retrospective analysis of data from the population‐based South Australian Cancer Registry (SACR). Data on all invasive CRC cases with the International Classification of Diseases for Oncology third edition (ICD‐O‐3) and primary site codes of C18‐C20 and C21.8, diagnosed between 1 January 1982 and 31 December 2017, were extracted from the SACR. The data were restricted to adults aged 20–89 who were identified with invasive CRC as their first cancer diagnosis. Individuals under 20 and over 89 were excluded, as CRC is rare in those under 20 [8, 19], and MPCs are underreported in those 90 and older [20, 21]. Cases were also excluded where there was another invasive cancer prior to the CRC diagnosis, primary colorectal sarcoma or lymphoma, CRC diagnosis recorded after the recorded date of death, CRC identified at autopsy, those deceased within 2 months of CRC diagnosis, or missing information on sex, age, date of diagnosis and histology. All eligible individuals were followed until 31 December 2019, for at least 2 years after the index CRC diagnosis unless they died, as indicated in previous studies [14, 22].

### 2.2 Definitions and Study Outcomes

In the current study, MPCs were defined as invasive neoplasms originating at a different anatomical site, or at the same site with distinct histology, which developed after the index CRC. When cancer occurred at the same site as the index CRC, the IACR/IARC rules and ICD‐O‐3 coding guidelines for behaviour, topography and morphology were followed to determine whether these could be considered as MPC [23, 24]. Non‐melanoma skin cancers, except for squamous cell carcinoma of the lip, genitalia, perineum and basal cell carcinoma of the genitalia and perineum, diagnosed after CRC were excluded, as SACR does not report these cancers. To minimise reporting bias, cancers diagnosed within the first 2 months following the diagnosis of the index CRC were excluded from being classified as MPCs [9, 25]. MPCs were classified as synchronous (cancers diagnosed within 2 to 6 months of the index CRC diagnosis) or metachronous (cancers diagnosed more than 6 months after the index CRC diagnosis). To identify and classify the tissue/site of the subsequent MPCs, the ICD‐O‐3 topography, histology and behaviour codes were considered (Supporting Information).

The primary outcome of this study was to determine the incidence of MPCs and whether individuals diagnosed with CRC have a higher risk of developing MPCs compared to the cancer risk in the general population of South Australia. The secondary outcomes included identifying the types of cancer that commonly arise as MPCs among CRC survivors and estimating the trend of MPCs over time.

### 2.3 Statistical Analysis

#### 2.3.1 Incidence of MPC

Time at risk for MPCs was determined from 2 months after the index diagnosis of CRC to the date of diagnosis of a subsequent primary cancer, death or the end of the follow‐up period (31 December 2019), whichever came first. In cases where an individual developed more than one subsequent MPC in different locations, the time at risk for each MPC was calculated independently as if it was the first MPC identified after the index CRC diagnosis, without considering any other intervening MPC(s) [14]. In contrast, for individuals who developed more than one cancer meeting the MPC definition at the same anatomical site, the time at risk was calculated up to the diagnosis of the first MPC, except in cases of haematological malignancies, where additional considerations may apply according to the IARC/IACR and ICD‐O‐3 rules of reporting multiple primaries [24, 26].

The cumulative incidence of MPCs in the presence of competing events was estimated using the *stcomlist* Stata module as described in Supporting Information (Data S1). The incidence rates were calculated by dividing the observed number of MPCs by the total number of individuals diagnosed with an index CRC and the corresponding total person‐years at risk, respectively, and reported per 100,000 population. Age‐ and sex‐standardisation was performed by an indirect standardisation method using cancer incidence in South Australia as the reference population parameter [27]. To account for the change in the population parameter over time, a 20‐year (2001–2020) average of age‐ and sex‐specific cancer incidence rates was used for standardisation (Supporting Information).

#### 2.3.2 Risk and Trends of MPC

The SIRs and absolute excess risks (AERs) were computed to estimate any excessive risk of MPC in individuals diagnosed with CRC compared to the risk in the general population of South Australia. The SIRs and their 95% confidence intervals (CIs) were computed under the assumption of Poisson distribution by dividing the observed number by the expected number of MPCs. Likewise, assuming a normal approximation of differences, AERs and their 95% CI were computed as the difference between the observed and expected numbers of MPCs divided by the person‐years at risk and multiplied by 10,000 [20]. The SIRs and AERs were stratified according to the following characteristics: age, sex, time since diagnosis of index CRC, and location/segment of colorectum where the index CRC was located. To determine whether common cancers influenced the MPC estimate, we also computed separate SIRs and AERs after excluding subsequent prostate and breast cancers from being considered as MPC, respectively. To evaluate the robustness of the estimates and assess whether surveillance bias and ascertainment criteria influenced the SIR estimate, as well as to examine the consistency of the estimates across different cut‐off times used to define MPCs, we performed a sensitivity analysis by excluding subsequent CRC and applying cut‐offs at 0 and 2 months after the diagnosis of the index CRC. The analyses were conducted with Stata version 18 (StataCorp, Texas).

The trend of MPC was assessed using the Joinpoint regression model (National Cancer Institute Joinpoint software, Windows Command‐line version 5.2.0.), with stratification of the ASR based on sex and the location index of CRC [28]. The Joinpoint model was fitted to the ASR of MPCs and tested for significant changes using the Monte Carlo permutation method [28]. The analysis assesses the changes in trends, the annual percentage changes (APCs) and the average APCs in ASR between joinpoints. The model estimates the variation for each joinpoint and follows the Poisson regression assumption. A *p*‐value of < 0.05 indicates significant changes in the trend of MPC between joinpoints. Results are described as increased or decreased for statistically significant changes in trends, and stable for non‐significant trends. To ensure the reliability of estimates, we restricted the trend analysis to the period 1990 to 2017. The number of index CRC cases diagnosed each year since 1990 was sufficient to reliably estimate the trends of MPCs with a low standard error. The trend was analysed in R software version 4.4.1 using the *nih.joinpoint* R package [29].

## 3 Results

### 3.1 Characteristics of the Study Population

The SACR included data on 36,402 individuals diagnosed with CRC from 1982 to 2017. After assessing study eligibility, 9673 were excluded for various reasons, resulting in a final cohort size of 26,729 for further analyses (Figure S1). Of these, 2252 (8.4%) were diagnosed before the age of 50 years, 14,260 (53.3%) were male, and 17,187 (64.3%) had colon cancer as the index cancer. The median age at index CRC diagnosis was 69 years (IQR: 60–77 years), and the median follow‐up time was 5.1 years (IQR: 2.1–11.0 years; Table 1). Based on the histological coding (ICD‐O‐3), 26,065 (97.5%) CRC cases were classified as an adenocarcinoma.

**TABLE 1:** Characteristics of cases with an index colorectal cancer diagnosis: Data from the South Australian Cancer Registry (1982–2017).

Variable		Frequency	Percent
Sex	Male	14,260	53.4
	Female	12,469	46.6
Age at CRC diagnosis, year	20–49	2252	8.4
	50–64	7676	28.7
	≥ 65	16,801	62.9
Location of CRC	Right‐sided colon	9245	34.6
	Left‐sided colon	7942	29.7
	Rectum	9542	35.7
Socio‐economic status (quintile)	Lowest	5207	19.5
	Low	5353	20
	Middle	5705	21.4
	High	5120	19.1
	Highest	5339	20

*Note:* Socioeconomic status is the socioeconomic index for areas (SEIFA) reported by the Australian Bureau of Statistics, which ranks areas into quintiles based on their relative socioeconomic advantages and disadvantages.

Abbreviation: CRC, colorectal cancer.

### 3.2 Incidence of MPC

**FIGURE 1:**
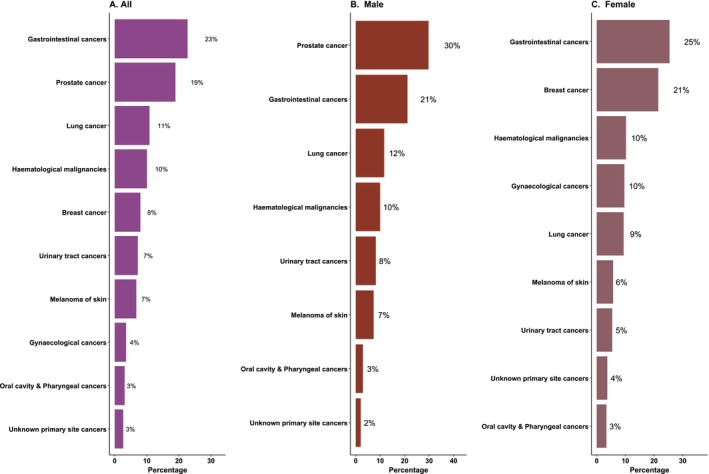
Common types of multiple primary cancers by sex in individuals diagnosed with colorectal cancer between 1982 and 2017.

During 191,895 person‐years of follow‐up, 3917 (14.7%) cases were diagnosed with at least one subsequent MPC, of which 467 (1.7%) individuals developed two or more subsequent MPCs at least 2 months after the diagnosis of the index CRC. Among 3917 individuals, 4453 MPCs were diagnosed, including 193 (4.3%) synchronous and 4260 (95.7%) metachronous. The median follow‐up time until the diagnosis of the first MPC was 6.4 years (IQR: 2.8–11.3 years). The cumulative incidence of MPCs in the presence of a competing event was 22.5% (95% CI: 21.6–23.4) for both sexes combined, 26.7% (95% CI: 25.3–28.2) in males and 18.0% (95% CI: 16.8–19.2) in females. The most common MPCs reported were prostate cancer (18.9%), subsequent CRC (13.1%), lung cancer (10.8%), haematological malignancies (10.2%), breast cancer (8.0%), urinary tract organ cancers (7.1%) and skin melanoma (6.7%), which collectively accounted for more than two‐thirds of all the MPCs. Gastrointestinal (GI) cancers, including oesophageal, gastric, small intestine, large intestine, liver, pancreatic, gallbladder and biliary tract cancers, were the most common group of related cancers, comprising 22.7% of all MPC diagnoses (Table 2 & Figure 1).

**TABLE 2:** Crude and age‐ and sex‐adjusted rate of multiple primary cancers in individuals diagnosed with index colorectal cancer: Data from the South Australian cancer registry (1982–2017).

Type of MPC	All		Males		Females	
	Crude rate (95% CI)	ASR (95% CI)	Crude rate (95% CI)	ASR (95% CI)	Crude rate (95% CI)	ASR (95% CI)
All MPCs	2040.3 (1977.4, 2105.2)	627 (607.7, 648)	2540 (2442, 2642.8)	693.3 (666.4, 721.2)	1530.4 (1453.8, 1611.1)	527.7 (502, 555.5)
Prostate cancer	—	—	866 (809.4, 926.5)	226.3 (211.3, 242.1)	—	—
Female breast cancer	—	—	—	—	366.7 (330.2, 407.3)	164.3 (147.6, 182.5)
Subsequent CRC	302.2 (278.6, 327.8)	84.4 (77.7, 91.5)	327.6 (293.5, 365.6)	88.3 (78.9, 98.6)	276.3 (244.9, 311.8)	78.1 (69, 88.2)
Lung cancer	250.8 (229.3, 274.2)	66.1 (60.3, 72.2)	338.9 (304.2, 377.6)	81.6 (73, 90.9)	160.8 (137.2, 188.4)	46.3 (39.3, 54.3)
Melanoma of skin	156.2 (139.5, 174.9)	57.6 (51.3, 64.5)	213.4 (186.2, 244.5)	69.7 (60.5, 79.9)	97.8 (79.8, 119.8)	41.1 (33.2, 50.4)
Urinary tract cancers	164.9 (147.7, 184.1)	45.7 (40.8, 51.0)	236.7 (208, 269.4)	61.8 (54.1, 70.4)	91.5 (74.1, 112.8)	26.6 (21.3, 32.8)
Bladder cancer	85.9 (73.7, 100)	20.2 (17.2, 23.5)	130.8 (109.9, 155.6)	29.6 (24.7, 35.3)	40.0 (29.1, 55.0)	9.7 (6.9, 13.4.8)
Renal cancer	64.6 (54.2, 77)	22.3 (18.5, 26.6)	90.8 (73.7, 111.9)	29.9 (24.0, 36.8)	37.9 (27.3, 52.5)	14.0 (9.8, 19.4)
Other urinary tract cancers	16.1 (11.4, 22.9)	4.0 (2.7, 5.7)	17.5 (10.9, 28.2)	4.6 (2.7, 7.4)	14.7 (8.7, 24.8)	3.8 (2.1, 6.4)
Gynaecological cancers	—	—	—	—	161.9 (138.3, 189.6)	64.8 (54.9, 75.9)
Corpus uteri cancer	—	—	—	—	86.2 (69.4, 107.1)	33.3 (26.5, 41.4)
Ovarian cancer	—	—	—	—	47.3 (35.3, 63.4)	18.2 (13.3, 24.3)
Cervical cancer	—	—	—	—	6.3 (1.8, 14.1)	4.6 (1.6, 10.0)
Other gynaecological cancers	—	—	—	—	25.2 (16.9, 37.7)	8.2 (5.2, 12.2)
Gastrointestinal cancers	516.2 (485.1, 549.4)	131.5 (123.6, 140.5)	604.4 (557.5, 655.3)	146.8 (135.3, 159.7)	426.2 (386.7, 469.8)	109.8 (103.1, 121.1)
Gastric cancer	50 (40.9, 61)	13.8 (11.1, 16.8)	75.3 (59.8, 94.7)	19.8 (15.5, 25.0)	24.2 (16.1, 36.4)	7.0 (4.4, 10.5)
Small intestinal cancer	18.2 (13.1, 25.4)	6.3 (4.4, 8.8)	20.6 (13.3, 32)	7.3 (4.5, 11.3)	15.8 (9.5, 26.2)	5.8 (3.2, 9.6)
Oesophageal cancer	31.3 (24.3, 40.2)	8.5 (6.6, 11.1)	41.3 (30.3, 56.3)	11.1 (8.1, 15.2)	21.0 (13.6, 32.6)	5.2 (3.4, 8.2)
Pancreatic cancer	67.2 (56.5, 79.8)	17.5 (14.8, 20.9)	85.6 (69.0, 106.1)	21.8 (17.6, 27.0)	48.4 (36.2, 64.6)	12.7 (9.6, 16.9)
Liver and intrahepatic bile duct	28.6 (22.0, 37.3)	8.7 (6.7, 11.4)	38.2 (27.7, 52.8)	11.4 (8.2, 15.7)	18.9 (11.9, 30.1)	5.3 (3.4, 8.4)
Gallbladder and bile duct cancer	25.0 (18,8, 33.2)	5.9 (4.5, 7.9)	21.7 (14.1, 33.2)	5.0 (3.3, 7.6)	28.4 (19.5, 41.4)	7.0 (4.8, 10.3)
Soft and connective tissue cancers	35.4 (27.9, 44.9)	9.8 (7.7, 12.5)	52.6 (40.0, 69.2)	13.2 (10.1, 17.3)	17.9 (11.1, 28.8)	5.6 (3.5, 9.0)
Cancers of retroperitoneum and peritoneum	5.2 (2.8, 9.7)	1.6 (0.9, 2.9)	4.1 (1.5, 11.0)	1.2 (0.5, 3.3)	6.3 (2.8, 14.1)	2.1 (0.9, 4.6)
Brain cancer	26.6 (20.2, 35.0)	10.6 (8.1, 13.9)	36.1 (25.9, 50.3)	13.7 (9.8, 19.1)	16.8 (10.3, 27.5)	6.9 (4.2, 11.3)
Thyroid cancer	10.9 (7.1, 16.8)	7.5 (4.9, 11.5)	14.4 (8.6, 24.4)	7.1 (4.2, 11.9)	7.4 (3.5, 15.4)	6.2 (3.2, 13.0)
Oral cavity and pharynx	71.8 (60.8, 84.9)	26.1 (22.1, 30.8)	85.6 (69.0, 106.1)	31.7 (25.6, 39.4)	57.8 (44.4, 75.3)	18.3 (14.0, 23.8)
Laryngeal cancer	8.9 (5.5, 14.2)	2.8 (1.8, 4.5)	16.5 (10.1, 26.9)	4.8 (2.9, 7.8)	1.1 (0.2, 7,5)	0.3 (0.1, 2.5)
Ocular cancer	6.8 (3.9, 11.7)	2.6 (1.5, 4.5)	9.3 (4.8, 17.8)	3.5 (1.8, 6.7)	4.2 (1.6, 11.2)	1.8 (0.7, 4.8)
All leukaemias	83.8 (71.8, 97.8)	26.3 (22.4.3, 30.7)	107.2 (88.5, 129.9)	32.4 (26.5, 39.3)	60 (46.2, 77.7)	20 (15.1, 25.9)
Chronic lymphocytic leukaemia	40.6 (32.5, 50.7)	11.5 (9.1, 14.4)	55.7 (42.7, 72.7)	15.7 (11.8, 20.4)	25.2 (16.9, 37.7)	7.9 (5.0, 11.7)
Acute myeloid leukaemia	29.7 (22.9, 38.5)	9.5 (7.2, 12.3)	38.2 (27.7, 52.7)	11.6 (8.2, 16.0)	21.0 (13.6, 32.6)	7.5 (4.6, 11.6)
Multiple myeloma	33.9 (26.6, 43.2)	9.4 (7.4, 12.0)	40.2 (29.4, 55.1)	10.6 (7.8, 14.6)	27.4 (18.6, 40.2)	7.9 (5.4, 11.6)
Myelodysplastic syndrome	25.0 (18.8, 33.2)	5.6 (4.2, 7.4)	37.1 (26.8, 51.5)	7.9 (5.6, 10.9)	12.6 (7.2, 22.2)	2.9 (1.7, 5.2)
Lymphomas	90.6 (78.1, 105.1)	30.2 (26.0, 35.1)	106.2 (87.5, 128.8)	33.7 (27.9, 40.9)	74.7 (59.2, 94.2)	25.8 (20.5, 32.5)
Hodgkin lymphoma	4.2 (2.1, 8.3)	3.3 (1.7, 6.6)	6.2 (2.8, 13.8)	4.7 (2.1, 10.1)	2.1 (0.5, 8.4)	1.8 (0.5, 7.2)
Non‐Hodgkin lymphoma	86.4 (74.2, 100.6)	27.3 (23.5, 31.7)	100.0 (82.0, 122.0)	30.2 (24.7, 36.8)	72.6 (57.3, 91.9)	23.7 (18.9, 29.9)
Unknown primary site cancers	61.4 (51.3, 73.6)	14.8 (12.4, 17.7)	59.8 (46.2, 77.4)	14.4 (11.2, 19.6)	63.1 (49.0, 81.2)	14.9 (11.6, 19.2)
All cancers excluding subsequent CRC	1821 (1761, 1883)	571.1 (552.4, 590.9)	2316 (2222, 2415)	635.1 (609.2, 662.1)	1314 (1242, 1389)	468.9 (443.4, 495.9)
All cancers excluding prostate cancer	1644.6 (1588.9, 1702.3)	536.6 (512.2, 562.1)	1751.4 (1671.9, 1834.7)	511.4 (488.2, 535.7)	1530.4 (1453.8, 1611.1)	558.4 (532.8, 589.2)
All cancers excluding breast cancer	1872.1 (1812, 1934.1)	554.2 (536.5, 572.6)	2540.4 (2442, 2642.8)	693.3 (666.4, 721.2)	1199.4 (1132.2, 1270.6)	407 (384.2, 431.2)

*Note:* A dash (—) indicates that the estimation does not apply to the specified group; All MPCs: included any cancers diagnosed after the index colorectal cancer that fulfil the criteria of MPC as defined in the method.

Abbreviations: ASR, age‐standardised rate; CI, confidence interval; MPCs, multiple primary cancers.

The ASR of MPC was 627.2 (95% CI: 607.9, 648) for both sexes combined, 693.3 (95% CI: 666.4, 721.2) for males and 527.7 (95% CI: 502, 555.5) for females (Table 2). Prostate cancer (ASR = 226.2, 95% CI: 211.3, 242.1), GI cancers (ASR = 146.8, 95% CI: 135.3, 159.7), lung cancer (ASR = 81.6, 95% CI: 73.0, 90.9) and skin melanoma (ASR = 69.7, 95% CI: 60.5, 79.9) were common MPC in males, while breast cancer (ASR = 164.3, 95% CI: 147.6, 182.5), GI cancers (ASR = 109.8, 95% CI: 103.1, 121.1), gynaecological cancers (ASR = 64.8, 95% CI: 54.9, 75.9) and lung cancer (ASR = 46.3, 95% CI: 39.3, 54.3) were found to be most common among females (Table 2). The incidence of MPC based on the follow‐up time since the index CRC diagnosis has also been presented in Table S1.

### 3.3 Risk of MPCs

The study results showed that individuals diagnosed with index CRC had a significantly higher risk of developing MPC compared to the risk of cancer in the general population with an SIR of 1.12 (95% CI: 1.09, 1.16). This corresponds to an additional 22.6 cancer cases per 10,000 population. CRC survivors were also found to have a significantly increased risk for particular tissue/site‐specific cancers, including subsequent CRC (SIR = 1.13, 95% CI: 1.04, 1. 22), lung cancer (SIR = 1.23, 95% CI: 1.12, 1.34), melanoma of skin (SIR = 1.31, 95% CI: 1.17, 1.46), urinary tract cancers (SIR = 1.42, 95% CI: 1.27, 1.58), leukaemias (SIR = 1.39, 95% CI: 1.19, 1.62), gastric cancer (SIR = 1.30, 95% CI: 1.06, 1.58), small intestinal cancer (SIR = 2.24, 95% CI: 1.61, 3.12), pancreatic cancer (SIR = 1.20, 95% CI: 1.02, 1.43) and oral cavity and pharyngeal cancer (SIR = 1.32, 95% CI: 1.12, 1.56) (Table 3 & Figure S2). When limited to extracolonic sites, the risk of non‐CRC MPC was still elevated compared to the corresponding risk of cancer in the general population with a SIR of 1.11 (95% CI: 1.08, 1.15; AER = 18.8, 95% CI: 16.9, 20.9); however, this significant elevation in risk was primarily in males (SIR = 1.10, 95% CI: 1.06, 1.15; AER = 21.4, 95% CI: 18.5, 24.5), while it was marginally significant in females (SIR = 1.05, 95% CI: 1.00, 1.10). Similarly, when excluding subsequent prostate cancer, a notable increase in the risk of MPC was observed among all CRC survivors (SIR = 1.09, 95% CI: 1.06, 1.13) and in male survivors (SIR = 1.10, 95% CI: 1.05, 1.15; Table 3 & Figure S2). The sensitivity analysis results were presented in the Supporting Information (Table S2).

**TABLE 3:** Risk of multiple primary cancers in individuals diagnosed with index colorectal cancer: Data from the South Australian Cancer Registry (1982–2017).

Type of MPC	All				Males				Females			
	Obs	Exp	SIR (95% CI)	AER (95% CI)/10,000	Obs	Exp	SIR (95% CI)	AER (95% CI)/10,000	Obs	Exp	SIR (95% CI)	AER (95% CI)/10,000
All MPCs	3917	3484	**1.12 (1.09, 1.16)**	22.6 (20.5, 24.8)	2462	2233	**1.10 (1.07, 1.15)**	23.6 (20.7, 26.9)	1455	1337	**1.09 (1.03, 1.14)**	12.4 (10.3, 14.9)
Prostate cancer	—	—	—	—	842	715	**1.18 (1.10, 1.26)**	13.1 (10.9, 15.5)	—	—	—	—
Female breast cancer	—	—	—	—	—	—	—	—	349	309	**1.13 (1.03, 1.26)**	4.2 (3.0, 5.7)
Gastrointestinal cancers	994	856	**1.16 (1.09, 1.24)**	7.1 (6.0, 8.4)	588	516	**1.14 (1.05, 1.24)**	7.4 (5.8, 9.3)	406	363	**1.12 (1.02, 1.23)**	4.5 (3.3, 6.1)
Gastric cancer	96	74	**1.30 (1.06, 1.58)**	1.2 (0.7, 1.7)	73	52	**1.41 (1.12, 1.77)**	2.2 (1.3, 3.3)	23	25	0.91 (0.61, 1.38)	−0.2 (−0.8, −0.03)
Small intestinal cancer	35	16	**2.24 (1.61, 3.12)**	1.0 (0.6, 1.6)	20	9	**2.23 (1.44, 3.46)**	0.1 (0.1. 0.2)	15	7	**2.19 (1.32, 3.63)**	0.8 (0.4, 1.7)
Oesophageal cancer	60	49	1.23 (0.96, 1.60)	0.6 (0.3, 1.0)	40	37	1.10 (0.80, 1.5)	0.4 (0.1, 1.1)	20	15	1.38 (0.90, 2.15)	0.5 (0.2, 1.2)
Pancreatic cancer	129	107	**1.20 (1.02, 1.43)**	1.2 (0.7, 1.7)	83	57	**1.45 (1.17, 1.80)**	2.7 (1.8, 3.9)	46	51	0.90 (0.68, 1.20)	−0.5 (−1.2, −0.2)
Liver and intrahepatic bile duct cancer	55	49	1.13 (0.87, 1.47)	0.3 (0.2, 0.7)	37	37	1.00 (0.72, 1.38)	—	18	14	1.28 (0.81, 2.03)	0.4 (0.1, 1.1)
Gallbladder and bile duct cancer	48	35	**1.37 (1.03, 1.82)**	0.7 (0.4, 1.2)	21	16	1.31 (0.86, 2.01)	0.5 (0.2, 1.2)	27	19	1.42 (0.98, 2.10)	0.8 (0.4, 1.7)
Subsequent CRC	581	515	**1.13 (1.04, 1. 22)**	3.4 (2.7, 4.4)	318	248	**1.28 (1.15, 1.43)**	7.2 (5.6, 9.1)	263	277	**1.16 (1.04, 1.31)**	3.8 (2.7, 5.3)
Lung cancer	482	393	**1.23 (1.12, 1.34)**	4.6 (3.7, 5.7)	329	263	**1.25 (1.12, 1.40)**	6.8 (5.32, 8.76)	153	142	1.08 (0.92, 1.26)	1.2 (0.6, 2.1)
Urinary tract cancers	317	224	**1.42 (1.27, 1.58)**	4.8 (3.9, 5.9)	230	168	**1.37 (1.21, 1.56)**	6.4 (4.9, 8.2)	87	66	**1.31 (1.06, 1.62)**	2.2 (1.4, 3.4)
Bladder cancer	165	114.9	**1.45 (1.24, 1.69)**	2.7 (2.0, 3.5)	127	92	**1.38 (1.16, 1.65)**	3.6 (2.5, 5.0)	38	28	1.36 (0.99, 1.87)	1.1 (0.5, 1.9)
Renal cancer	124	90	**1.38 (1.16, 1.65)**	1.8 (1.2, 2.5)	88	65	**1.37 (1.11, 1.68)**	2.4 (1.5, 3.6)	36	29	1.25 (0.90, 1.73)	0.7 (0.3, 1.5)
Other urinary tract cancers	31	20	**1.53 (1.08, 2.17)**	0.6 (0.3, 1.0)	17	11	1.53 (0.95, 2.46)	0.6 (0.2, 1.4)	14	9	1.50 (0.88, 2.51)	0.5 (0.2, 1.2)
All leukaemias	161	116	**1.39 (1.19, 1.62)**	2.3 (1.7, 3.1)	104	75	**1.39 (1.15, 1.68)**	3.0 (2.0, 4.3)	57	44	1.29 (0.99, 1.67)	1.4 (0.7, 2.3)
Chronic lymphocytic leukaemia	78	51	**1.52 (1.22, 1.90)**	1.4 (0.9, 2.1)	54	32	**1.70 (1.30, 2.21)**	2.3 (1.4, 3.4)	24	18	1.33 (0.90, 2.0)	0.6 (0.3, 1.4)
Acute myeloid leukaemia	57	34	**1.69 (1.31, 2.20)**	1.2 (0.8, 1.8)	37	22	**1.70 (1.23, 2.34)**	1.6 (0.9, 2.6)	20	13	**1.57 (1.01, 2.43)**	0.7 (0.3, 1.5)
Multiple myeloma	65	56	1.16 (0.91, 1.48)	0.3 (0.2, 0.6)	39	35	1.13 (0.83, 1.55)	0.4 (0.1, 1.1)	26	23	1.16 (0.79, 1.70)	0.3 (0.1, 0.9)
Myelodysplastic syndrome	48	44	1.09 (0.82, 1.44)	0.2 (0.1, 0.5)	36	30	1.21 (0.87, 1.67)	0.6 (0.2, 1.4)	12	16	0.77 (0.44, 1.35)	−0.2 (−0.8, −0.03)
Lymphomas	174	166	1.05 (0.90, 1.22)	0.4 (0.2, 0.8)	103	99	1.04 (0.86, 1.26)	0.4 (0.1, 1.1)	71	70	1.02 (0.81, 1.28)	0.1 (0.03, 0.6)
Non‐Hodgkin lymphoma	166	156	1.07 (0.92, 1.24)	0.5 (0.3, 1.0)	97	93	1.05 (0.86, 1.28)	0.4 (0.2, 1.1)	69	65	1.06 (0.84, 1.34)	0.4 (0.1, 1.1)
Hodgkin lymphoma	8	7	1.21 (0.61, 2.42)	0.05 (0.01, 0.3)	6	4	1.47 (0.66, 3.27)	0.2 (0.03, 01)	2	3	0.75 (0.20, 3.01)	−0.1 (−0.6, −0.03)
Gynaecological cancers	—	—	—	—	—	—	—	—	154	117	**1.32 (1.13, 1.55)**	3.9 (2.7, 5.4)
Uterine cancer	—	—	—	—	—	—	—	—	82	60	**1.36 (1.10, 1.69)**	2.3 (1.5, 3.5)
Ovarian cancer	—	—	—	—	—	—	—	—	45	31	**1.45 (1.08, 1.94)**	1.5 (0.8, 2.5)
Cervical cancer	—	—	—	—	—	—	—	—	6	8.2	0.73 (0.33, 1.63)	−0.2 (−0.8, −0.3)
Other gynaecological cancers	—	—	—	—	—	—	—	—	24	17	1.41 (0.95, 2.10)	0.7 (0.3, 1.5)
Soft and connective tissue cancers	68	66	1.02 (0.81, 1.30)	0.2 (0.1, 0.4)	51	51	0.99 (0.76, 1.13)	—	17	18	0.93 (0.58, 1.49)	−0.2 (−0.6, −0.03)
Cancers of retroperitoneum and peritoneum	10	12	0.83 (0.45, 1.55)	−0.1 (−0.4, −0.01)	4	3	1.23 (0.46, 3.29)	—	6	8	0.72 (0.32, 1.60)	−0.2 (−0.8, −0.03)
Brain cancer	49	39	1.26 (0.95, 1.67)	0.5 (0.3, 0.9)	32	24	1.34 (0.96, 1.89)	1.0 (0.5, 1.9)	16	15	1.04 (0.64, 1.70)	0.1 (0.03, 0.6)
Thyroid cancer	21	20	1.01 (0.66, 1.55)	0.1 (0.02, 0.3)	14	8	1.67 (0.99, 2.83)	0.6 (0.2, 1.4)	7	12	0.59 (0.3, 1.23)	−0.5 (−1.2, −0.2)
Oral cavity and pharyngeal cancer	136	104	**1.30 (1.10, 1.54)**	1.7 (1.1, 2.4)	81	72	1.13 (0.91, 1.40)	0.9 (0.4, 1.8)	55	37	**1.50 (1.15, 1.95)**	1.9 (1.1, 3.0)
Laryngeal cancer	17	15	1.12 (0.70. 1.81)	0.10 (0.01, 0.4)	16	15	1.08 (0.66, 1.77)	0.1 (0.03, 0.6)	1	2	0.57 (0.1, 4.1)	−0.1 (−0.6, −0.03)
Ocular cancer	13	9	1.46 (0.85, 2.52)	0.2 (0.01, 0.5)	9	5	1.83 (0.95, 3.52)	0.4 (0.1, 1.1)	4	4	1.01 (0.38, 2.68)	—
Melanoma of skin	300	230	**1.31 (1.17, 1.46)**	3.6 (2.8, 4.6)	207	153	**1.36 (1.18, 1.56)**	5.6 (4.2, 7.32)	93	84	1.11 (0.91, 1.36)	1.0 (0.4, 1.8)
Unknown primary site cancers	115	138	0.84 (0.70, 1.02)	−1.2 (−1.8, −0.8.4)	58	73	0.78 (0.60, 1.02)	−1.7 (−2.7, −0.9)	60	66	0.88 (0.68, 1.14)	−0.8 (−1.7, −0.4)
All cancers excluding subsequent CRC	3439	3084	**1.11 (1.08, 1.15)**	18.8 (16.9, 20.9)	2212	2008	**1.10 (1.06, 1.15)**	21.4 (18.5, 24.5)	1227	1165	1.05 (1.00, 1.11)	6.5 (5.0, 8.4)
All cancers excluding prostate cancer	3236	2967	**1.09 (1.06, 1.15)**	13.7 (12.1, 15.4)	1781	1626	**1.10 (1.05, 1.15)**	15.2 (13.0, 17.8)	1455	1337	**1.09 (1.03, 1.14)**	12.4 (10.3, 14.9)
All cancers excluding breast cancer	3617	3324	**1.09 (1.05, 1.12)**	15.2 (13.5, 17.1)	2462	2233	**1.10 (1.07, 1.15)**	23.6 (20.7, 26.9)	1155	1042	**1.11 (1.05, 1.18)**	11.7 (9.7, 14.1)

*Note:* Bold numbers indicate a significant increase compared to the expected numbers in the general population; A dash (—) indicates that the estimation does not apply to the specified group; All MPCs: included any cancers diagnosed after the index CRC that fulfil the criteria of MPC as defined in the method; Bold numeric values indicate a significantly elevated risk of MPC beyond the expected incidence in the general population.

Abbreviations: AER, absolute excess risk; CI, confidence interval; CRC, colorectal cancer; Exp, expected number of MPCs; MPCs, multiple primary cancers; Obs, observed number of MPC cases; SIR, standardised incidence ratio.

Analysis at 1, 2, 3, 5, 10 and 15 years after the diagnosis of the index CRC revealed an increased risk of MPC at each respective follow‐up time point. For male survivors, the risk remained consistently high throughout the follow‐up time, while for female survivors, the risk significantly increased after 5 years post‐diagnosis of the index CRC, although it was marginally significant in the first year of the follow‐up period (Table S3).

Among young‐onset CRC survivors who were < 50 years old at the time of index CRC diagnosis, the risk of developing MPC was 52% higher than the general population (SIR = 1.52, 95% CI: 1.32, 1.75), with an AER of 31.4 cases per 10,000 population (AER = 31.4, 95% CI: 24.4, 39.8). In CRC survivors who were ≥ 50 years old at index CRC diagnosis, the risk of developing MPC was 11% higher than the general population (SIR = 1.11, 95% CI: 1.07, 1.15; AER = 21.4 cases per 10,000 population). There was also a marked increase in MPC risk for survivors of both colon and rectal cancer compared to the general population. In males, the MPC risk was significantly higher for survivors of both colon and rectal cancer, while in females, rectal but not colon cancer survivors had a higher risk of developing a subsequent MPC (Table 4 & Table S4).

**TABLE 4:** Risk of multiple primary cancers by sex, age and anatomical location of the index colorectal cancer.

MPC by participant characteristics	All				Males				Females			
	Obs	Exp	SIR (95% CI)	AER (95% CI)/10,000	Obs	Exp	SIR (95% CI)	AER (95% CI)/10,000	Obs	Exp	SIR (95% CI)	AER (95% CI)/10,000
Age at CRC diagnosis												
< 50 years	200	131	**1.52 (1.32, 1.75)**	31.4 (24.4, 39.8)	109	69	**1.57 (1.30, 1.90)**	35.7 (25.3, 48.7)	91	61	**1.50 (1.22, 1.84)**	27.9 (18.8, 39.9)
≥ 50 years	3717	3352	**1.11 (1.07, 1.15)**	21.4 (19.3, 23.8)	2353	2164	**1.09 (1.05, 1.13)**	22.0 (19.0, 25.4)	1364	1277	**1.07 (1.02, 1.13)**	10.3 (8.3, 12.7)
Location of CRC												
Colon	2539	2257	**1.13 (1.08, 1.17)**	23.3 (20.6, 26.2)	1553	1367	**1.14 (1.08, 1.20)**	32.5 (28.0, 37.5)	986	915	**1.08 (1.01, 1.15)**	11.1 (8.7, 14.0)
Right‐sided colon	1322	1197	**1.11 (1.05, 1.17)**	19.9 (16.6, 23.7)	731	644	**1.14 (1.06, 1.22)**	32.3 (25.9, 39.9)	591	530	**1.11 (1.03, 1.21)**	17.0 (13.0, 21.9)
Left‐sided colon	1217	1060	**1.15 (1.09–1.21)**	26.9 (22.8, 31.4)	822	723	**1.14 (1.06, 1.22)**	32.9 (26.8, 40.0)	395	385	1.03 (0.93, 1.13)	3.6 (1.8, 6.4)
Rectum	1378	1227	**1.12 (1.07, 1.18)**	21.3 (18.1, 25.0)	909	867	1.05 (0.98, 1.11)	10.6 (7.7, 14.3)	469	422	**1.11 (1.01, 1.22)**	15.1 (11.1, 20.0)
Socio‐economic status												
Lowest level	713	626	**1.14 (1.06, 1.23)**	24.9 (19.9, 30.7)	463	402	**1.15 (1.05, 1.26)**	34.3 (26.3, 44.1)	250	239	1.05 (0.92, 1.18)	6.4 (3.2, 11.5)
Low level	763	657	**1.16 (1.08, 1.25)**	29.0 (23.8, 35.1)	490	432	1.14 (1.04, 1.24)	30.6 (23.2, 39.5)	273	245	**1.12 (0.99, 1.25)**	16.0 (10.6, 23.1)
Middle level	906	775	**1.17 (1.10, 1.15)**	31.3 (26.2, 37.1)	561	484	**1.16 (1.07, 1.26)**	37.0 (29.2 46.3)	345	304	**1.13 (1.02, 1.26)**	19.2 (13.8, 26.0)
High level	744	692	1.08 (1.00, 1.16)	13.9 (10.3, 18.2)	472	442	1.07 (0.98, 1.17)	15.8 (10.7, 22.6)	272	260	1.05 (0.93, 1.18)	6.5 (3.3, 11.3)
Highest level	790	745	1.06 (0.99, 1.14)	11.1 (8.1, 14.8)	476	474	1.01 (0.92, 1.10)	1. 0 (0.1, 3.6)	314	290	1.08 (0.97, 1.21)	11.8 (7.6, 17.5)

*Note:* Bold numbers indicate a significant increase compared to the expected numbers in the general population.

Abbreviations: AER, absolute excess risk; CI, confidence interval; Exp, expected number of MPCs; MPC, multiple primary cancer; Obs, observed number of MPCs; SIR, standardised incidence ratio.

### 3.4 Trends of MPC Over Time

**FIGURE 2:**
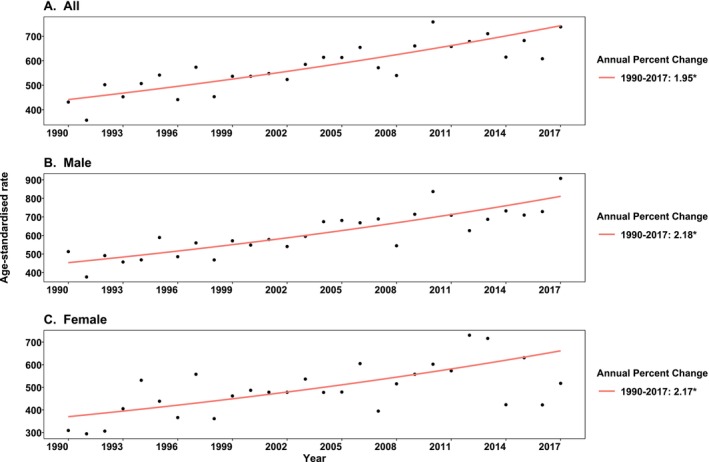
Trends of multiple primary cancers in individuals diagnosed with colorectal cancer in South Australia between 1990 and 2017. (A) Presented APC of MPC trend in both sexes combined, including all site cancers; (B) Presented APC of MPC trend in males excluding male breast cancer; (C) Presented APC of MPC trend in females.

An analysis of trends in MPCs over time revealed that the ASR increased by 1.95% annual change from 1990 to 2017 (APC = 1.95, 95% CI: 1.33, 2.51). There was a statistically significant increase in the incidence of MPCs in both males (APC = 2.18, 95% CI: 1.54, 2.75) and females (APC = 2.17, 95% CI: 1.02, 3.18) during the follow‐up period (Figure 2). Moreover, the trend of MPC incidence exhibited a statistically significant increase in both individuals diagnosed with index colon cancer (APC = 2.20, 95% CI: 1.24, 2.76) and rectal cancer (APC = 1.56, 95% CI: 0.63, 2.37) (Figure S3).

## 4 Discussion

In this study, 14.7% of CRC survivors were diagnosed with a MPC at a median of 6.4 years after the index CRC. They also had a 12% higher risk of developing subsequent MPCs compared with the expected risk of any cancer diagnosis in the general population. Even when limiting to extra‐colonic sites after excluding subsequent CRCs from being considered MPCs, the risk of developing other subsequent primary cancers remains significantly elevated by 11% (95% CI: 8%–13%) compared to the general population, highlighting that CRC survivors have a higher risk of subsequent cancers beyond CRC, independent of surveillance effects. Prostate cancer, subsequent CRC, lung cancer, breast cancer, leukaemia, lymphoma, urinary tract cancers and skin melanoma were the most common cancers identified as MPCs, which collectively constituted over two‐thirds of the MPCs in CRC survivors. The incidence of MPC has significantly increased over time, with an average annual rise of 1.95% between 1990 and 2017. While many studies have reported an elevated risk of subsequent cancers in CRC survivors [8, 11, 12, 14], others have found no such increase [17, 18, 30]. To our knowledge, this is the first study to use contemporaneous data to evaluate the risk of subsequent cancers among CRC survivors in Australia.

Previous studies have shown a high risk of subsequent cancers in CRC survivors, including GI, female genital, breast, prostate, urinary tract, lung cancers, melanoma and haematological malignancies [8, 11, 12, 14]. Consistent with these studies, our results revealed an elevated risk for these cancers. The increased risk of subsequent cancers after CRC diagnosis may result from the multifactorial nature of carcinogenesis and shared risk factors. Many cancers share common modifiable and non‐modifiable risks, with 42% linked to poor lifestyle factors such as smoking, obesity, alcohol use, inactivity and poor diet [31]. Genetic, hormonal and treatment‐related factors also play a role. Contrary to our findings, some studies have reported no increased risk of subsequent primary cancers in CRC survivors [17, 18, 30]. This discrepancy may arise from variations in participant selection criteria, outcome definitions, length of follow‐up time, and genetic backgrounds. For instance, Tanaka et al. (2021) analysed data of CRC patients who had undergone surgical resection from three clinical trials [30]. This method likely excluded high‐risk patients, potentially leading to an underestimation of the risk of subsequent cancers.

The current study found that CRC survivors have an increased risk of developing GI cancers, which share common risk factors with CRC, such as genetic mutations, smoking, alcohol use, obesity, sedentary behaviour and metabolic abnormalities [32]. Altered gut and oral microbiota, contributing to chronic inflammation, further heighten this risk by promoting DNA damage, stem cell proliferation and metaplasia, particularly in colorectal, gastric and oesophageal cancers [33]. Inflammatory mediators, genetic mutations and reactive oxygen species contribute to the development of subsequent GI cancers. Subsequent CRC was the most common GI cancer, likely due to the persistent susceptibility or exposure of colorectal mucosa to genetic, inflammatory and environmental and behavioural risk factors, even after tumour removal.

This study found a significantly elevated incidence of prostate cancer among male CRC survivors. This may be attributed to both cancers being age‐related and sharing common risk factors such as diet, smoking, obesity and metabolic syndrome‐related conditions, suggesting a bidirectional relationship where the occurrence of one cancer increases the risk of the other [34, 35]. In addition, shared genetic susceptibility may play a role, as a genome‐wide association study identified significant associations between single nucleotide polymorphisms in five regions of chromosome 8q24 and elevated risks for both colon and prostate cancers [36]. Similarly, in female CRC survivors, the risk of breast cancer was significantly higher, consistent with previous studies [12, 14]. Both cancers are among the most commonly diagnosed cancers in Australia, and the evaluated risk could be linked to overlapping risk factors. This may partly be attributed to shared genetic predispositions, including mutations in genes like *BRCA1*, *BRCA2*, *MLH1* and *MLH2*, which are associated with susceptibility to both cancers [37].

In this study, CRC survivors showed a significantly elevated risk of haematological malignancies, although the literature on this is inconsistent. Some studies report no significant increase in risk [12, 14], while others find higher leukaemia incidence in CRC survivors [38]. One study even reported a low risk of lymphatic and haematopoietic malignancies in colon cancer survivors [8]. Conversely, Lee et al. (2015) found an increased risk of haematological malignancies in colon cancer survivors, but not in rectal cancer survivors [12]. These inconsistencies may be due to changes in histological classifications over time [39]. Moreover, neoadjuvant and adjuvant chemotherapy for solid cancers has been linked to therapy‐related complications, including leukaemogenesis [40] and radiation for rectal cancer can affect the haematopoietic system [41]. A previous study showed that chemotherapy‐treated patients had a higher risk of therapy‐related acute myeloid leukaemia and myelodysplastic syndrome in most solid cancers, including rectal/rectosigmoid cancer, but not colon cancer [40]. This supports our finding of an increased leukaemia risk in rectal cancer survivors compared to the general population.

The data from the current study indicate a higher risk of developing any type of MPC in both men and women. However, the absolute risk assessment reveals a two‐fold increase in MPC risk in males compared to females, with an AER of 23.6 and 12.4 cases per 10,000 population for men and women, respectively. This disparity may reflect the generally higher incidence of cancer in men, as documented in epidemiological studies, where males typically exhibit a higher cancer risk than females for most non‐gender‐specific anatomical sites [42]. Several physiological, genetic, epigenetic and immunologic factors, including testosterone levels, may contribute to this increased risk in men. A high level of testosterone is thought to promote cell growth and tumorigenesis, whereas higher levels of progesterone and oestrogen in women are linked to a lower risk for certain non‐sex‐specific cancers [43, 44]. Additionally, behavioural and environmental factors, such as smoking, alcohol consumption, obesity and poor diet, which increase cancer risk, are more prevalent in men than in women [45].

The risk of MPCs increases regardless of the age at which CRC is diagnosed. However, CRC survivors diagnosed before age 50 have a 52% higher risk of developing an MPC compared to an 11% risk in those diagnosed at 50 and beyond. The findings highlight an elevated risk across all age groups but a more pronounced risk in early‐onset CRC survivors. For survivors over 50, the increased MPC risk likely reflects age‐related cancer susceptibility [22]. Several factors may contribute to an elevated risk in younger survivors. Early‐onset CRC is strongly linked to hereditary predisposition and family history [46]. Additionally, early‐onset CRC is associated with distinct molecular features, including epigenetic changes suggestive of global DNA hypomethylation, and a high proportion of these patients have a disease classified as molecular‐1 subtype, which is characterised by high microsatellite instability, chronic inflammation and expression of immunogenic markers [47]. Dysfunctional DNA repair, immune regulation and apoptosis are involved in most forms of cancer; therefore, these features may collectively drive the increased susceptibility to MPCs in younger CRC survivors.

Specific anatomical locations of the index CRC increased the risk of MPCs. In our study, both colon and rectal cancers were linked to an elevated risk of specific subsequent cancers, with variations based on sex. Males with colon cancer and females with colon and rectal cancer showed a higher MPC risk compared to the general population, potentially due to differences in treatment strategies. This risk difference in colon and rectal cancer survivors with respect to sex might be attributed to the treatment approaches. Rectal cancer is commonly managed with neoadjuvant therapy and radiation before surgery [8], which increases the risk of uterine, cervical, ovarian and bladder cancers in females but reduces the risk of prostate cancer [48]. On the other hand, colon cancer is often treated with adjuvant chemotherapy after surgery [8]. This aligns with our findings, which revealed a higher incidence of genital organ cancers among females with rectal cancer, while no increased risk of prostate cancer was observed in males with rectal cancer. Our study also showed that right‐sided colon cancers were associated with a higher MPC risk in both sexes compared to left‐sided colon and rectal cancers, where increased risk was observed only in males. Proximal (right‐sided) colon cancers exhibit distinctive clinicopathological and molecular features, including larger tumour size, higher grade and mucinous histology, as well as frequent *BRAF* mutations and a high microsatellite instability phenotype [49]. These tumours also express tumour‐associated genetic markers (e.g., *CD44v6, CDX2, BRAF*) and immune markers (e.g., CD68, CD163), alongside characteristics like CpG island methylator phenotype and *KRAS* mutations [50]. These features are linked with defective DNA mismatch repair (hereditary or sporadic) and epigenetic dysregulation, which can predispose patients to additional cancers, both within the colorectum and in extracolonic sites. In addition, due to its anatomical position, the right‐sided colon might have experienced a longer exposure to luminal contents and bile acids, potentially promoting chronic inflammation and DNA damage [51, 52], which likely increases the risk of subsequent cancers. Moreover, right‐sided colon cancer tends to present at advanced stages due to its subtle symptoms [53], which may promote field effects in the mucosa that likely increase the risk of metachronous CRC [54]. Furthermore, because of the high recurrence and metastasis commonly associated with advanced stages of the disease [55], patients with right‐sided colon cancer may undergo more extensive post‐treatment surveillance, potentially identifying MPCs more frequently than survivors of left‐sided colon cancer and rectal cancer.

The overall risk of MPCs remained significantly elevated throughout the 15‐year follow‐up period after the diagnosis of index CRC. Interestingly, sex‐based differences were observed in the temporal pattern of MPC development. In males, the incidence of MPCs showed a consistent increase over the entire 15‐year period, indicating a continuous need for vigilant monitoring. In contrast, females exhibited a delayed elevation in MPC risk, with a notable increase emerging 5 years after the index CRC diagnosis. Evidence also suggests that the incidence of MPC is marginally higher among male cancer survivors compared to females throughout their survivorship [9], primarily due to lifestyle‐ and behaviour‐related risk factors [56]. However, for female cancer survivors, the risk of developing MPC increases with longer survival time [57]. These findings highlight the importance of personalised follow‐up and surveillance strategies for CRC survivors, tailored to individual risk profiles based on factors such as sex and the time elapsed since Index CRC diagnosis. Such strategies can enhance the prevention, early detection and management of subsequent cancers, thereby significantly improving outcomes for CRC survivors.

From 1990 to 2017, we observed an increasing trend in the incidence of MPCs in both sexes. This trend may be attributed to awareness of cancer‐related symptoms among the population, the implementation of cancer surveillance programmes, and frequent follow‐up and monitoring of cancer survivors, all of which contribute to higher detection rates of subsequent primary cancers [22]. In support of this argument, though not specific to CRC survivors, a systematic review reported that the incidence of MPCs has increased in the USA and Australia since the 1980s [58]. The Global Burden of Disease Study (1990–2019) highlights a rising burden of cancer risk factors, including lifestyle, environmental, occupational, behavioural and metabolic factors [59]. For CRC survivors, these factors, such as diets high in processed meats or fast foods and low in fibre—known to increase the risk of recurrence and subsequent CRC—smoking, lack of physical activity, excessive alcohol consumption and prolonged exposure to environmental toxins, carcinogens or ionising radiation, may drive MPCs by promoting inflammation, oxidative stress and DNA damage [60, 61, 62].

The current study has certain limitations that should be considered when interpreting the findings. Firstly, the registry data did not include information on cancer staging and treatment details. As a result, the study could not assess how the risk of MPCs varies based on treatment and clinical profiles, such as tumour stage or specific treatment modalities like surgery, chemotherapy or radiation. Secondly, while a 2‐month interval between the diagnosis of the index CRC and subsequent MPCs was applied to minimise misclassification and reporting bias, the possibility of incorrectly classifying certain metastatic and recurrent cancers as MPCs cannot be entirely ruled out. In addition, the standardisation of incidence rates and SIRs was performed using an average age‐ and sex‐specific cancer incidence rate, derived across calendar years, as a proxy for calendar‐year‐specific age‐ and sex‐specific incidence rates. This approach assumes a stable background cancer risk over time, which might not fully account for temporal variations in incidence due to calendar‐year‐specific factors. This approximation was adopted due to the unavailability of calendar‐specific incidence data prior to 2001 during the study period; however, it might introduce some bias in the standardised estimates compared to those obtained using calendar‐year‐specific rates. Furthermore, cancer survivors often have more frequent physician visits and follow‐up than the general population, which may lead to increased detection of subsequent cancers and might introduce surveillance bias. Therefore, future research should consider adjusting for the frequency of post‐cancer diagnosis follow‐up when estimating the risk of MPCs to ensure more accurate comparisons. Despite these limitations, this study provides valuable evidence regarding the overall risk of subsequent primary cancers, as well as cancer‐specific risks for the majority of tissue types or sites among CRC survivors. The study also utilised large, state‐wide population‐based cancer registry data collected over more than three decades, enabling capture of long‐term trends and providing a substantial basis for understanding the burden of MPCs in CRC survivors. These findings make a significant contribution to the growing body of literature on survivorship in cancer care.

In conclusion, this study revealed that CRC survivors are at an increased risk of developing subsequent primary cancers compared to the general population. The most common MPCs identified were prostate cancer, subsequent CRC, lung cancer, haematological malignancies, breast cancer, urinary tract organ cancers and skin melanoma, with the risk of these cancers notably higher among CRC survivors. Moreover, the incidence of MPC has shown an increasing trend over time in both males and females. Therefore, understanding the risk of MPCs could help inform strategies to enhance and refine surveillance programmes for early detection of subsequent cancers, potentially improving treatment outcomes and overall survival.

## Author Contributions

Mulugeta Melku: Conceptualisation; methodology; investigation; formal analysis; project administration; resources; software; validation; visualisation; writing – original draft; writing – revising and editing. Erin L. Symonds: Conceptualisation; methodology; investigation; software; project administration; resource; data curation; supervision; validation; visualisation; writing – review and editing. Oliver G. Best: Conceptualisation; methodology; investigation; software; resources; supervision; validation; visualisation; data curation; writing – review and editing. Jean M. Winter: Conceptualisation; methodology; investigation; resources; supervision; validation; visualisation; data curation; writing – review and editing. Lauren A. Thurgood: Conceptualisation; methodology; investigation; resources; supervision; validation; visualisation; data curation; writing – review and editing. Ganessan Kichenadasse: Conceptualisation; methodology; investigation; validation; visualisation; writing – review and editing. Muktar Ahmed: Methodology; investigation; data analysis; visualisation; validation; resources; software; writing – review and editing. Murthy Mittinty: Methodology; investigation; data analysis; visualisation; validation; writing – review and editing. Molla M. Wassie: Methodology; investigation; data analysis; visualisation; validation; resources; software; writing – review and editing.

## Ethics Statement

The study was approved by the South Australian Department for Health and Wellbeing Human Research Ethics Committee (Ref: 2022/HRE00169) and was conducted in accordance with the principles, regulations and ethical conduct of research prescribed by the Australian Government and its regulatory authorities.

## Conflicts of Interest

The authors declare no conflicts of interest.

## Supporting information


Data S1.

## Data Availability

The data used in this study were obtained from the South Australian Cancer Registry (SACR). The authors accessed the data under a data use agreement with SACR. The data that support the findings of this study are available in the manuscript and Supporting Information. Upon request, additional information can be obtained from the corresponding authors.
