# Supplementary material for: Incidence, Risk and Trends of Multiple Primary Cancers in Patients With Colorectal Cancer: Evidence From the South Australian Cancer Registry
Source: Cancer Med. 2025 May 30;14(11):e70984. doi: 10.1002/cam4.70984 (PMC12123453; doi:10.1002/cam4.70984)
Supplement: Supplementary file 1 — Data S1. [file CAM4-14-e70984-s001.docx]

**SUPPLEMENTARY MATERIALS**

**Incidence, risk, and trends of multiple primary cancers in patients with colorectal cancer: evidence from the South Australian Cancer Registry**

Mulugeta Melku^1,2^, Oliver G Best^1^, Jean M Winter^1^, Lauren A Thurgood^1^, Muktar Ahmed^1^, Ganessan Kichenadasse^1,3^, Murthy Mittinty^1^, Molla M Wassie^1^, Erin L Symonds^1,4^

^1^Flinders Health and Medical Research Institute, College of Medicine and Public Health, Flinders University, South Australia, Australia

^2^Department of Hematology and Immunohematology, School of Biomedical and Laboratory Sciences, College of Medicine and Health Science, University of Gondar, Ethiopia

^3^Medical Oncology Department, Flinders Centre for Innovation in Cancer, Flinders Medical Centre, South Adelaide Local Health Network, Bedford Park, South Australia, Australia

^4^Gastroenterology Department, Flinders Medical Centre, South Adelaide Local Health Network, Bedford Park, South Australia, Australia

**CORRESPONDING AUTHORS:**

Mulugeta Melku, [gobe0011@flinders.edu.au](mailto:gobe0011@flinders.edu.au).

1. **SUPPLEMENTARY METHODS**
   1. **Study design, setting and population**

This study was a population-based retrospective analysis of data from the South Australian Cancer Registry (SACR). The SACR operates under the South Australian Department for Health and Wellbeing and has been collecting data concerning cancer incidence and mortality since 1977. The primary sources of information for the SACR include reports from pathology laboratories, hospitals, radiotherapy departments, the registry of births, deaths, and marriages (BDM), and other supplementary sources such as clinicians.

Data on all invasive CRC cases with the International Classification of Diseases for Oncology third edition (ICD-O-3) and primary site codes of C18-C20 and C21.8, diagnosed between 1 January 1982 and 31 December 2017, were extracted from the SACR. The data were restricted to adults aged 20-89 who were identified with invasive CRC as their first cancer diagnosis. We excluded individuals <20 and >89 years of age, as CRC is uncommon before the age of 20,^1, 2^ and MPCs in people 90 years or over are underreported.^3, 4^ Cases were also excluded where there was invasive cancer prior to the CRC diagnosis, the histology was reported as CRC with sarcoma and lymphoma (ICD-O-3), the CRC diagnosis was recorded after their recorded date of death, the CRC was identified solely by autopsy, the individual died within two months of their CRC diagnosis, or if there was missing information on sex, age and date of diagnosis and histology. All eligible individuals were followed until December 31, 2019, for at least two years after the index CRC diagnosis, unless they died, as indicated in previous literature.^5, 6^

- 1. **Definitions and study outcomes**

In the current study, MPCs were defined as invasive neoplasms originating at a different anatomical site, or at the same site with distinct histology, which developed after the index CRC. When cancer occurred at the same site as the index CRC, the International Association of Cancer Registries (IACR)/International Agency for Research on Cancer (IARC) rules and ICD-O-3 coding guidelines for behaviour, topography and morphology were followed to determine whether these could be considered as MPC.^7, 8^ Non-melanoma skin cancers (except for squamous cell carcinoma of the lip, genitalia and perineum, and basal cell carcinoma of the genitalia and perineum) diagnosed after CRC diagnosis were included as the SARC does not report these cancers. To minimise reporting bias, cancers diagnosed within the first two months following the diagnosis of the index CRC were excluded from being classified as MPCs.^9, 10^ MPCs were classified as synchronous (cancers diagnosed within two to six months of the index CRC diagnosis) or metachronous (cancers diagnosed more than six months after the index CRC diagnosis).

To identify and classify the tissue/site of the subsequent MPC, ICD-O-3 topography, histology and behaviour codes were considered. Two key variables, “Sequence Number Central” and “Unique Identification Number,” were used to identify individuals with MPCs. Each tissue/site-specific subsequent cancer was identified and classified in the dataset, where the sequence number central was two and above after CRC diagnosis. Furthermore, for the estimation of tissue- or site-specific MPC, higher-order cancers (with sequence number central three and above) were counted as MPC. This approach enables comparison of the observed site- or tissue-specific MPC in the cohort with the expected numbers in the general population because the SACR reports by consolidating multiple primaries into a single primary per individual, in accordance with the site and histology specifications of IACR rules to ensure the comparability of cancer data over time.^11, 12^

The primary outcome of this study was to determine the incidence of MPCs and whether individuals diagnosed with CRC have a higher risk of developing MPCs compared to the cancer risk in the general population of South Australia. The secondary outcomes included identifying the types of cancer that commonly arise as MPCs among CRC survivors and estimating the trend of MPCs over time.

- 1. **Statistical analysis**
     1. **Incidence of MPC**

Person-year at risk (PY) for MPCs was accumulated from two months after the index diagnosis of CRC (2 months lapse time) to the date of diagnosis of subsequent primary cancers, death, or the end of the follow-up period (December 31, 2019), whichever came first. In cases where an individual developed more than one subsequent MPCs in different locations, the time at risk for each MPC was calculated independently as if it was the first MPC identified after the index CRC diagnosis, without considering any other intervening MPC(s).^5^ In contrast, for individuals who developed more than one cancer meeting the MPC definition at the same anatomical site, the time at risk was calculated up to the diagnosis of the first MPC, except in cases of haematological malignancies, where additional considerations may apply according to the IARC/IACR and ICD-O-3 rules of reporting multiple primaries.^8, 12^

The overall cumulative incidence of MPCs was calculated using the cumulative incidence function (CIF) with the Stata *stcomlist* command in a competing risks framework, treating death before MPC as the competing event. Each time an MPC occurred, the cause-specific hazard of MPC was estimated as the number of MPC cases divided by the number of individuals still at risk (i.e., alive and without an MPC). This hazard was multiplied by the overall survival probability, representing the proportion of individuals free of both MPC and death just before that time. The CIF was then computed by summing these contributions across all relevant time points up to a specified time, yielding the probability of developing an MPC while accounting for the competing risk of death. To express this probability as a percentage, the cumulative incidence was multiplied by 100.. In addition, overall and site-specific MPC incidence rates of MPCs were calculated by dividing the observed number of MPCs by the corresponding total person-years at risk and reported per 100,000 population as follows:

r*_ij_*  = ∑O_ij_/PY_ij_  X100,000 where,

r*_ij_* denotes the incidence rate of MPC j in age group *i* during the person-years PY*_ij_*.

O_ij_ denotes the observed number of cases for MPC 𝑗 in age group *i* during the person-years PY*_ij_*.

PY_ij_ denotes the person-years at risk of developing MPC *j* in age group *i.*

Age-sex-standardisation of rates was performed by an indirect standardisation method using cancer incidence in South Australia as reference population parameters. As age- and sex-specific cancer incidence data were not available or accessible during this study, a 20-year (2001-2020) average of age- and sex-specific cancer incidence rates was used to account for the change in the population parameter (incidence of cancer) over time.^13^ The incidence of MPCs was then adjusted using the following equation:

……${ASR}_{\left( ind \right)j}=\left( {\sum\text{O}}_{\text{ij}}/R_{ij}{PY}_{ij} \right)*\text{R}_{\text{j}}$

Where:

O_ij_ denotes the observed cases for MPC *j* in age group *i* of the study population or the total sex-specific cases of MPC j in age group *i* for the study population for that specific sex

$R_{ij}$ denotes the age- and sex-specific cancer incidence rate in age group i for cancer j in the standard population*.*

${PY}_{ij}$ denotes person-year at risk in age group *i* for cancer *j* in the study population.

R_j_ denotes crude cancer incidence rate or sex-specific crude cancer incidence rate for the cancer *j in* the standard population, expressed per 100,000 population.

ASR_(ind)j_  denotes the age-standardised (age- and sex-standardised) rate of MPC *j* using indirect standardisation method

In brief, the ASR was calculated by multiplying the person-year at risk for events in each age group of 5-year intervals in our cohort by the average age- and sex-specific cancer incidence of the corresponding age groups in the South Australian population. These values were then summed to obtain the expected number of MPCs. The crude cancer incidence rate per 100,000 people in the reference population was multiplied by the observed-to-expected MPC ratio to get the ASR ^14^.

- - 1. **Estimation of risk of MPCs**

The overall and tissue- or site-specific SIRs were calculated as the ratio of observed-to-expected number of MPC cases as O*_ij_*/E*_ij_*, where E_ij_ denotes the expected number for MPC *j* during the PY*_ij_*.^15^ The SIRs and their 95% confidence intervals (CIs) were computed under the assumption of Poisson distribution by dividing the observed number by the expected number of MPCs. Likewise, assuming a normal approximation of difference, the absolute excess risks (AERs) and their 95% CI were computed as the difference between the observed and expected numbers of MPCs divided by the person-years at risk and multiplied by 10,000 ((O*_ij_* - E*_ij_*) X10,000/PY*_ij_*).^16, 17^ For males, females and both sexes combined, the expected number of MPCs was estimated as follows:

$$E_{ij}=\sum R_{ij}{PY}_{ij}$$

Where:

E_ij_ denotes the sex-specific or both sexes combined expected number of cases in age group *I* for MPC 𝑗 during the person-years PY*_ij_*.

$R_{ij}$ denotes age- and sex-specific cancer incidence rate in age group *i* for cancer *j* in the standard population

${PY}_{ij}$ denotes person-year at risk in age group *i* for MPC *j* in the study population

The SIRs and AERs were stratified according to the following characteristics: age, sex, time since diagnosis of index CRC and location/segment of colon/rectum where the index CRC was located.

To determine whether common cancers influenced the MPC estimate, we also computed separate SIRs and AERs after excluding prostate and breast cancer from being considered as MPCs, respectively. Given that prostate and breast cancer are the most prevalent cancers among males and females, respectively, in Australia, the sensitivity analysis was conducted by excluding subsequent prostate and breast cancers, while keeping the cohort of CRC survivors the same. This enabled investigation into whether the observed increase in MPCs among CRC survivors was driven solely by the elevated incidence of prostate and breast cancers or if it reflected a broader increased risk for other subsequent cancers.^18^ In addition, to check whether post-CRC surveillance influenced the risk of MPCs, we estimated a separate SIR excluding subsequent CRCs. The analyses were conducted with Stata version 18 (StataCorp, Texas).

- - - 1. **Sensitivity analysis**

Two sensitivity analyses were conducted to assess the robustness of the estimates. First, we excluded cancers diagnosed within the first two months following the index CRC diagnosis to minimise reporting bias. Second, we analysed the data by including all cancers diagnosed either synchronously with or after the index CRC diagnosis.^19, 20^ We also conducted a sensitivity analysis defining MPCs as cancers diagnosed six months after the index CRC diagnosis, focusing on the overall MPCs and common site/tissue-specific MPC types identified among the study participants.

- - 1. **Estimation of trends of MPCs**

The trend of MPCs was assessed using the Joinpoint regression model in National Cancer Institute Joinpoint software (Windows Command-line version 5.2.0.), with stratification of the ASR based on sex and the location index of CRC.^21^ The Joinpoint model was fitted to the ASR of MPCs and tested for significant changes using the Monte Carlo permutation method.^21^ The analysis assesses the changes in trends, the APCs and the average annual percentage changes (AAPC) in ASR between joinpoints. The model estimates the variation for each joinpoint and follows the Poisson regression assumption.^22^ The model tests the null hypothesis that APC or AAPC are zero.^22^ A p-value of less than 0.05 indicates significant changes in the trend of MPC between joinpoints. Results are described as increased or decreased for statistically significant changes in trends, and stable for non-significant trends.^20^ To ensure the reliability of estimates, we restricted the trend analysis to the period 1990 to 2017 as the number of index CRC cases diagnosed each year since 1990 was sufficient to reliably estimate the trends of MPCs with a low standard error. The trend was analysed in R software version 4.4.1 using *nih.joinpoint* R package.

- 1. **Ethical considerations**

The study was carried out in adherence to the principles of the "Declaration of Helsinki," Good Clinical Practice (GCP), the National Statement on Ethical Conduct in Human Research (NHMRC, 2007), the Australian Code for the Responsible Conduct of Research (2007), and within the laws and regulations of Australia. Ethics approval was obtained from the South Australian Department for Health and Wellbeing Human Research Ethics Committee (2022/HRE00169). Once data access is granted, non-identifiable datasets provided by SACR are analysed in accordance with the principles, regulations, and ethical conduct of research prescribed by the Australian Government and its regulatory authorities.

**Supplementary methods Table 1**: Classification tissue-or site-specific MPC types based on ICD-O-3 topography and histology codes

| **Cancer types** | **ICD-O-3 topography** | **ICD-O-3 Histology** | **Remarks** |
| --- | --- | --- | --- |
| Oral cavity and Pharynx | C000 – C14.8 | Any histology except 9120, 9755-9758, 9140 and 9590-9992 |  |
| Larynx | C32.0-C32.9 |  |  |
| Oesophagus | C15.0-C15.9 |  |  |
| Stomach | C16.0-C16.9 |  |  |
| Small intestine | C17.0-C17.9 |  |  |
| Colorectum | C18.0-C18.9, C19.9, C20.9, C21.8 | Any histology except 9120, 9755-9758, 9140 and 9590-9992 | Subsequent primary CRC at a different site or the same site with different histology from the index CRC were considered MPC |
| Other digestive organs (Retroperitoneum, peritoneum, anus, anal canal and other ill-defined digestive system) | C21.0-C21.2, C26.0-C26.9, C48.0-48.8 | Any histology except 9120, 9755-9758, 9140 and 9590-9992 |  |
| Liver and intrahepatic bile duct | C22.0-C22.1 |  |  |
| Gallbladder and biliary tract | C24.0-C24.9 |  |  |
| Pancreas | C25.0-C25.9 |  |  |
| Lung and bronchus | C34.0-C34.9 |  |  |
| Nasal cavity, middle ear Nose, accessory sinus, trachea and other ill-defined respiratory system | C30.0-C31.9, C33.9, C39.0-C39.9 |  |  |
| Soft tissues (heart, mediastinum and pleura), and Connective, subcutaneous and other soft tissues | C38.0-C38.9, C439.0-C49.9) | Any histology except 9590-9992 |  |
| Bone, joint and cartilage | C40.0-0C41.9 | Any histology except 9120, 9755-9758, 9140 and 9590-9992 |  |
| Breast | C50.0-C50.9 |  |  |
| Cervix Uteri | C53.0-53.9 |  |  |
| Corpus Uteri | C54.0-54.9, C55.9 |  |  |
| Ovary | C56.9 |  |  |
| Volva, Vagina, and other unspecified female genital organs | C51.0-51.9, C52.9, C57.1-57.9, C58.9 |  |  |
| Prostate | C61.9 |  |  |
| Male genital organ | C60.0-C63.9 |  |  |
| Kidney | C64.9 |  |  |
| Bladder | C67.0-C67.9 |  |  |
| Renal Pelvis and other Urinary tract organs cancer | C65.9, C66.9 & C68.0-68.9 |  |  |
| Eye and Orbit | C69.0-C69.9 |  |  |
| Brain and central nervous system | C70.0-C72.9 |  |  |
| Thyroid | C73.9 |  |  |
| Other Endocrine Organs | C74.0-C74.9, C75.0-C75.9 |  |  |
| Unknown primary sites | C80.9 |  |  |
| Melanoma of Skin | C44.0-C44.9 | 8720-8790 |  |
| Hodgkin Lymphoma |  |  |  |
| - Hodgkin-Nodal | C02.4, C09.8-C09.9, C11.1, C14.2, C37.9, C42.2, C77.0-C779 | 9650-9667 |  |
| - Hodgkin-extranodal | Any sites | 9650-9667 |  |
| Non-Hodgkin Lymphoma |  |  |  |
| - Non-Hodgkin Lymphoma – Nodal | C02.4, C09.8, C09.9, C11.1, C14.2, C37.9, C42.2, C77.0-  C77.9 | 9590-9597, 9670-9671, 9673, 9675, 9678-9680, 9684, 9687-9691, 9695, 9698-9702, 9705, 9708-9709, 9712, 9714-9719, 9724-9729, 9735, 9737-9738, *9811-9818*, *9823*, *9827*, *9837* |  |
| - Non-Hodgkin Lymphoma –Extranodal | All sites except C02.4, C09.8, C09.9, C11.1, C14.2, C37.9, C42.0, C42.1, C42.2, C42.4, C77.0-  C77.9 | 9590-9597, 9670-9671, 9673, 9675, 9678-9680, 9684, 9687, 9688, 9689-9691, 9695, 9698-9702, 9705, 9708-9709, 9712, 9714-9719, 9724-9729, 9735, 9737-9738 |  |
| Multiple Myeloma and plasma cell Tumours | C41.9, C42.0, C42.1 | 9731-9734, 9761 |  |
| All Leukaemias | C42.1 | 9800-9948 |  |
| Chronic lymphocytic leukaemia | C42.1 | 9823 |  |
| Acute lymphoblastic leukaemia | C42.1 | 9727-9729, 9811-9818, 9835-9837, |  |
| Acute Myeloid Leukaemia | C42.1 | *9840, 9861, 9865-9874, 9891, 9895-9897, 9910-9911, 9920* |  |
| Chronic myeloid leukaemia | C42.1 | 9863, 9875-9876, 9945-9946 |  |
| Myeloproliferative disorders | C42.1 | 9950-9967 |  |
| Other Haematological disorders | C42.1 | 9970-9975 |  |
| Myelodysplastic syndrome | C42.1 | *9980-9992* |  |

1. **SUPPLEMENTARY RESULTS**
   1. **Study participants' characteristics**

The SACR included data on 36,402 individuals diagnosed with CRC from 1982 to 2017. As Clearly described in Supplementary figure, after excluding cases with prior invasive cancer (n=4,497), unconfirmed diagnoses (n=2,880), primary colorectal lymphoma/leukaemia/sarcoma histology (n=104), age at CRC diagnosis <20 or >89 years old (n=802), individuals who died within two months of index CRC diagnosis (n=1,387) or cases where the date of death preceded the date of CRC diagnosis (n=3), the 26,729 of CRC cases were used for further analyses (Supplementary figure 1). Of these, 2,252 (8.4%) were diagnosed before the age of 50 years, 14,260 (53.3%) were male, and 17,187 (64.3%) had colon cancer as the index cancer. The median age at index CRC diagnosis was 69 years (IQR: 60-77 years), and the follow-up time was 5.1 years (IQR: 2.1-11.0 years) (Table 1).

- 1. **Risk of MPC by sex**

In males, the risk for all types of MPC was high (SIR = 1.10, 95% CI: 1.06, 1.15), with a significantly higher risk of developing prostate cancer (SIR = 1.18, 95% CI: 1.10, 1.26), subsequent CRC (SIR = 1.28, 95% CI: 1.15, 1.43), melanoma of the skin (SIR = 1.36, 95% CI: 1.18, 1.56), urinary tract organ cancers (SIR = 1.37, 95% CI: 1.21, 1.56), leukemia (SIR = 1.39, 95% CI: 1.15, 1.68), gastric cancer (SIR = 1.41, 95% CI: 1.12, 1.77), small intestinal cancer (SIR = 2.23, 95% CI: 1.44, 3.46), pancreatic cancer (SIR = 1.45, 95% CI: 1.17, 1.80), and brain cancer (SIR = 1.43, 95% CI: 1.02, 1.98). Likewise, a higher risk of MPC was also observed among female CRC survivors (SIR = 1.09, 95% CI: 1.03, 1.14). In females, breast cancer (SIR= 1.13, 95% CI: 1.03, 1.26), urinary tract organ cancer (SIR = 1.31, 95% CI: 1.06, 1.62), acute myeloid leukemia (SIR = 1.57, 95% CI: 1.01, 2.43), gynaecologic cancers (SIR = 1.32, 95% CI: 1.13, 1.55) including uterine cancer (SIR = 1.36, 95% CI: 1.10, 1.69) and ovarian cancer (SIR = 1.45, 95% CI: 1.08, 1.94), small intestinal cancer (SIR = 2.19, 95% CI: 1.32, 3.63), and oral cavity and pharyngeal cancers (SIR = 1.50, 95% CI: 1.15, 1.95) were all elevated compared to the general female population (Table 3 and Figure 2).

- 1. **Supplementary Tables**

**Supplementary Table 1:** Crude and age-sex-standardised rate of multiple primary cancers based on follow-up time after diagnosis of the index colorectal cancer

| **MPC by follow-up length since the index CRC diagnosis** | **All** | | **Males** | | **Females** | |
| --- | --- | --- | --- | --- | --- | --- |
|  | **Crude rate(95%CI)** | **ASR (95%CI)** | **Crude rate (95%CI)** | **ASR (95%CI)** | **Crude rate (95%CI)** | **ASR (95%CI)** |
| Overall | 2040.3 (1977.4, 2105.2) | 627 (607.7, 648) | 2540 (2442, 2642.8) | 693.3 (666.4, 721.2) | 1530.4 (1453.8, 1611.1) | 527.7 (502, 555.5) |
| MPC within 1st year | 1966.1 (1783.8, 2166.9) | 684.2 (620.8, 754.3) | 2383.2 (2110.6, 2688.9) | 755.6 (670.1, 850.8) | 1491.9 (1267.1, 1756.6) | 575.3 (488.4, 677.3) |
| MPC within the 2 years | 1822.4 (1697.3, 1956.8) | 630.5 (585.0, 677.2) | 2217.5 (2029.8, 2422.6 | 692.1 (633.5, 756.2) | 1373.9 (1218.9, 1548.7) | 527.6 (466.7, 594.3) |
| MPC within the 3 years | 1752.5 (1649.9, 1861.5) | 597.8 (562.8, 636.3) | 2111.8 (1958.3, 2277.2) | 672.8 (633.3,716) | 1346.7 (1218.0, 1488.9) | 511.3 (462.4, 565.2) |
| MPC within the 5 years | 1831 (1745.0, 1921.7) | 613.0 (584.4, 643.3) | 2213.6 (2084.2, 2351.0) | 666.9 (630.7, 709.6) | 1404.7 (1296.9, 1521.4) | 524.6 (486.7, 568.7) |
| MPC within the 10 years | 1932.4 (1861.3, 2006.1) | 624.2 (600.9, 647.6) | 2329.6 (2221.9, 2442.5) | 672.3 (650.4, 711.5) | 1502.8 (1413.4, 1597.7) | 543.0 (510.3, 578.9) |
| MPC within the 15 years | 1990.5 (1924.4, 2058.9) | 627.0 (607.1, 648.6) | 2457.2 (2355.2, 2563.7) | 689.9 (663.6, 722.7) | 1498.2 (1416.9, 1584.1) | - 1. (502.2, 559.4) |

ASR: Age-standardised rate; CI: confidence interval; CRC: Colorectal cancer; MPC: Multiple primary cancer

**Supplementary Table 2:** Risk of multiple primary cancer in individuals diagnosed with index colorectal cancer, including cancers diagnosed within 2 months of index colorectal cancer diagnosis as a sensitivity analysis: Data from the South Australian cancer registry (1982-2017)

| **Type of MPC** | **All** | | | | **Males** | | | | **Females** | | | |
| --- | --- | --- | --- | --- | --- | --- | --- | --- | --- | --- | --- | --- |
|  | **Obs** | **Exp** | **SIR (95%CI)** | **AER (95%CI) /10,000** | **Obs** | **Exp** | **SIR (95%CI)** | **AER (95%CI)/10,000** | **Obs** | **Exp** | **SIR (95%CI)** | **AER (95%CI)/10,000** |
| All MPCs | 4406 | 3501 | **1.26(1.22, 1.30)** | **46.7(43.7, 49.9)** | 2759 | 2240 | **1.23(1.19, 1.28)** | **53.1(48.7, 57.9)** | 1647 | 1346 | **1.22 (1.17, 1.28)** | 31.4(27.9, 35.1) |
| Prostate cancer | - | - | ***-*** | *-* | 884 | **729** | **1.21 (1.14,1.30)** | **15.6 (13.3, 18.3)** | - | - | **-** | - |
| Female breast cancer | - | - | **-** | - | - | - | **-** | - | 366 | 315 | **1.16 (1.05, 1.29)** | 5.3 (3.9, 6.9) |
| Gastrointestinal cancers | 1317 | 864 | **1.52 (1.44, 1.61)** | **23.2 (21.2, 25.5)** | 788 | 521 | **1.51 (1.41, 1.62)** | **27.1(24.0, 30.6)** | 529 | 366 | **1.45 (1.33, 1.57)** | 16.9 (14.4, 19.7) |
| Oesophageal cancer | 63 | 50 | 1.27 (0.99, 1.63) | 0.7 (0.4, 1.1) | 42 | 37 | 1.13 (0.83, 1.52) | 0.5 (0.2, 1.2) | 21 | 15 | 1.43 (0.93, 2.20) | 0.6(0.2, 1.4) |
| Gastric cancer | 103 | 76 | **1.36 (1.13, 1.66)** | 1.4 (0.9, 2.0) | 78 | 53 | **1.47 (1.18, 1.84)** | **2.5 (1.6, 3.7)** | 25 | 26 | 0.91 (0.61, 1.38) | -0.1 (-0.6, -0.03) |
| Pancreatic cancer | 133 | 109 | **1.22 (1.03, 1.44)** | 1.2 (0.8, 1.8) | 83 | 57 | **1.46 (1.18, 1.80)** | 2.7 (1.8, 4.0) | 48 | 52 | 0.92 (0.70, 1.23) | -0.4(-1.1, -0.1) |
| Liver and intrahepatic bile duct cancer | 59 | 50 | 1.19 (0.92, 1.53) | 0.5 (0.2, 0.9) | 40 | 38 | 1.06 (0.77, 1.44) | 0.2 (0.02, 0.7) | 19 | 14 | 1.33 (0.85, 2.08) | 0.5 (0.2, 1.2) |
| Subsequent CRC | 887 | 520 | **1.71(1.60, 1. 82)** | **18.9 (17.0, 20.9**) | 507 | 300 | **1.69 (1.55, 1.84)** | **20.9 (18.2, 24.0)** | 380 | 229 | **1.66 (1.50, 18.1)** | 15.7 (13.3, 18.4) |
| Lung cancer | 509 | 400 | **1.27 (1.17, 1.39)** | **5.6 (4.6, 6.7)** | 347 | 268 | **1.30 (1.17, 1.44)** | **8.0 (6.3, 10.0)** | 162 | 145 | 1.12 (0.96, 1.31) | 1.8 (1.0, 2.8) |
| Urinary tract cancers | 362 | 228 | **1.59 (1.43, 1.76)** | 6.8 (5.7, 8.1) | 262 | 171 | **1.54 (1.36, 1.74)** | 9.2 (7.4, 11.3) | 100 | 68 | **1.48 (1.22, 1.80)** | 3.3 (2.3, 4.7) |
| Bladder cancer | 175 | 116 | **1.51 (1.30, 1.75)** | 3.0 (2.3, 3.9) | 134 | 93 | **1.44 (1.21, 1.70)** | 4.1 (3.0, 5.6) | 41 | 29 | **1.44 (1.06, 1.96)** | 1.0 (0.5, 1.9) |
| All leukaemias | 174 | 118 | **1.48 (1.27, 1.71)** | 2.9 (2.2, 3.7) | 113 | 76 | **1.48 (1.23, 1.78)** | 3.7 (2.6, 5.1) | 61 | 45 | **1.36 (1.06, 1.74)** | 1.7 (0.9, 2.7) |
| Chronic lymphocytic leukaemia | 89 | 52 | **1.71 (1.39, 2.10)** | 1.9 (1.3, 2.6) | 89 | 52 | **1.71 (1.39, 2.10)** | 1.9 (1.3, 2.6) | 28 | 18 | **1.53 (1.05, 2.20)** | 1.0 (0.5, 1.9) |
| Acute myeloid leukaemia | 59 | 34 | **1.72 (1.33, 2.22)** | 1.3 (0.8, 1.9) | 39 | 22 | **1.75 (1.28, 2.39)** | 1.7 (1.0, 2.7) | 20 | 13 | 1.54 (1.00, 2.39) | 0.7 (0.3, 1.5) |
| Lymphomas | 203 | 169 | **1.20 (1.05, 1.38)** | 1.7 (1.2, 2.4) | 122 | 101 | **1.21 (1.01, 1.44)** | 2.1 (1.3, 3.2) | 81 | 71 | 1.14 (0.91, 1.42) | 1.0 (0.5, 1.9) |
| Non-Hodgkin lymphoma | 194 | 158 | **1.22 (1.06, 1.41)** | 1.8 (1.3, 2.5) | 115 | 95 | **1.22 (1.01, 1.46)** | 2.0 (1.2, 3.1) | 79 | 67 | 1.19 (0.95, 1.48) | 1.2 (0.6, 2.2) |
| Gynaecological cancers | - | - | - | **-** | - | - | - | **-** | 177 | 119 | **1.49 (1.29, 1.73)** | 6.0 (4.5, 7.7) |
| Uterine cancer | - | - | - | - | - | - | - | - | 86 | 62 | **1.40 (1.13, 1.73)** | 2.5 (1.6, 3.7) |
| Brain cancer | 50 | 40 | 1.26 (0.95, 1.66) | 0.5 (0.2, 0.9) | 34 | 25 | 1.36 (0.97, 1.88) | 0.9 (0.4, 1.7) | 16 | 17 | 1.02 (0.63, 1.67) | - 0.1 (-0.6, -0.03) |
| Oral cavity and pharyngeal cancer | 137 | 107 | **1.29 (1.09, 1.52)** | 1.5(1.0, 2.2) | 82 | 74 | 1.12 (0.90, 1.39) | 0.8 (0.4, 1.6) | 55 | 38 | **1.47 (1.13, 1.91)** | 1.8(1.0, 2.8) |
| Melanoma | 312 | 234 | **1.33 (1.19, 1.49)** | 3.9 (3.1, 5.0) | 214 | 156 | **1.38 (1.20, 1.57)** | 5.8 (4.4, 7.6) | 98 | 85 | 1.15 (0.94, 1.40) | 1.3 (0.7, 2.3) |
| Unknown primary site cancers | 128 | 140 | 0.91 (0.77, 1.09) | -0.6(-1.1, -0.3) | 61 | 74 | 0.82 (0.64, 1.06) | -1.3 (-2.2, -0.7) | 67 | 67 | 1.00 (0.79, 1.27) | - |
| All cancers excluding subsequent CRC | 3670 | 3179 | **1.16 (1.12, 1.19)** | 25.1 (23.0, 27.5) | 2353 | 2063 | **1.14 (1.10, 1.19)** | 29.4 (26.1, 33.0) | 1317 | 1204 | **1.09 (1.04, 1.16)** | 11.7 (9.6, 14.0) |

AER: Absolute excess risk; CI: confidence interval; CRC: Colorectal cancer; Exp: expected number of MPCs; MPCs: Multiple primary cancers; Obs: Observed number of MPC cases; SIR: Standardised incidence ratio; Bold numbers indicate a significant increase compared to the expected numbers in the general population; A dash ("-") indicates that the estimation does not apply to the specified group; All MPCs: included any cancers diagnosed after the index CRC that fulfil the criteria of MPC as defined in the method; Bold numeric values indicate a significantly elevated risk of MPC beyond the expected incidence in the general population. This is a sensitivity analysis to check the robustness of the estimate including cancer diagnosed within two months of index CRC diagnosis.

**Supplementary Table 3**: Risk of multiple primary cancers based on follow-up time since diagnosis of the index colorectal cancer

| **MPC by follow-up time since CRC diagnosis** | **All** | | | | **Males** | | | | **Females** | | | |
| --- | --- | --- | --- | --- | --- | --- | --- | --- | --- | --- | --- | --- |
|  | **Obs** | **Exp** | **SIR (95%CI)** | **AER (95%CI)/ 10,000** | **Obs** | **Exp** | **SIR (95%CI)** | **AER (95%CI)/10,000** | **Obs** | **Exp** | **SIR (95%CI)** | **AER (95%CI)/10,000** |
| MPC within first year | 406 | 329 | **1.23 (1.12, 1.36)** | 37.3 (29.4, 46.6) | 262 | 216 | **1.21 (1.08, 1.37)** | 41.8 (30.6, 55.8) | 144 | 121 | 1.18 (1.0, 1.39) | 22.8 (14.3, 34.5) |
| MPC within 2 years | 759 | 670 | **1.13 (1.06, 1.22)** | 21.4 (17.2, 26.3) | 491 | 441 | **1.11 (1.02, 1.22)** | 22.6 (16.8, 29.8) | 268 | 246 | 1.09 (0.97, 1.23) | 11.3 (7.1, 17.1) |
| MPC within the 3 years | 1056 | 980 | **1.08 (1.02, 1.15)** | 12.6 (9.9, 15.8) | 675 | 646 | **1.05 (1.01, 1.13)** | 9.1 (6.1, 13.0) | 381 | 360 | 1.06 (0.96, 1.17) | 7.4 (4.6, 11.4) |
| MPC within the 5 years | 1662 | 1505 | **1.10 (1.05, 1.16)** | 17.3 (14.7, 20.2) | 1059 | 991 | **1.07 (1.01, 1,14)** | 14.2 (11.0, 18.0) | 603 | 556 | **1.09 (1.02, 1.18)** | 11.0 (8.0, 14.6) |
| MPC within the 10 | 2,738 | 2442 | **1.12 (1.08, 1.16)** | 20.9 (18.6, 23.4) | 1715 | 1596 | **1.08 (1.03, 1.13)** | 16.2 (13.4, 19.3) | 1023 | 913 | **1.12 (1.05, 1.19)** | 16.2 (13.3, 19.5) |
| MPC within the 15 years | 3,369 | 2992 | **1.13 (1.09, 1.17)** | 222.7 (20.1, 24.6) | 2135 | 1942 | **1.10 (1.05, 1.15)** | 22.2 (19.2, 25.6) | 1234 | 1130 | **1.09 (1.03, 1.16)** | 12.6 (10.3, 15.3) |
| Overall | 3,917 | 3484 | **1.12 (1.09, 1.16)** | 22.6 (20.5, 24.8) | 2462 | 2233 | **1.10 (1.07, 1.15)** | 23.6 (20.7, 26.9) | 1455 | 1337 | **1.09 (1.03, 1,14)** | 12.4 (10.3, 14.9) |

AER: Absolute excess risk; CI: confidence interval; CRC: Colorectal cancer; Exp: expected number of MPCs; MPCs: Multiple primary cancer; Obs: Observed number of MPCs; SIR: Standardised incidence ratio; Bold numbers indicate a significant increase compared to the expected numbers in the general population

**Supplementary Table 4**: Risk of selected types of multiple primary cancers based on the location of index colorectal cancer

| **Type of MPC** | **Sex** | **Colon** | | | | **Rectum** | | | |
| --- | --- | --- | --- | --- | --- | --- | --- | --- | --- |
|  |  | **Obs** | **exp** | **SIR (95%CI)** | **AER (95%CI)/10,000** | **Obs** | **exp** | **SIR (95%CI)** | **AER (95%CI)/10,000** |
| Leukaemias | All | 96 | 75 | **1.27 (1.04, 1.56)** | 1.7 (1.1, 2.7) | 65 | 40 | **1.61 (1.27, 2.06)** | 3.5 (2.3, 5.2) |
|  | Male | 62 | 46 | **1.35 (1.05, 1.73)** | 2.8 (1.6, 4.5) | 42 | 29 | **1.45 (1.08, 1.97)** | 3.3 (1.8, 5.6) |
|  | Females | 34 | 31 | 1.12 (0.80, 1.56) | 0.5 (0.1, 1.4) | 23 | 14 | **1.67 (1.11, 2.52**) | 2.9 (1.8, 5.5) |
| Prostate cancer | Males | 563 | 433 | **1.30 (1.20, 1.41)** | 22.6 (18.9, 26.8) | 279 | 290 | 0.99 (0.88, 1.11) | -1.0 (-2.6, -0.3) |
| Gynaecological cancers | Female | 112 | 79 | **1.42 (1.18, 1.71)** | 5.2 (3.6, 7.3) | 42 | 29 | **1.46 (1.08, 1.97)** | 4.2 (2.2, 7.1) |

AER: Absolute excess risk; CI: confidence interval; Exp: expected number of MPC; MPC: Multiple primary cancer; Obs: Observed number of MPCs; SIR: Standardised incidence ratio; Bold numbers indicate a significant increase compared to the expected numbers in the general population

- 1. **Supplementary Figures**

**Supplementary Figure 1**: Flowchart of study participant selection

Cases diagnosed with CRC from 1982-2017 (N = 36,402)

Excluded (n = 9,673)

- Prior invasive cancer (n = 4,497)
- Unconfirmed diagnosis (n=2,880)
- Deceased within 2 months of index CRC diagnosis (n = 1,387)
- Age at index CRC diagnosis <20 or >89 (n = 802)
- Primary colorectal lymphoma/leukemia/sarcoma (n = 104)
- Unknown age at index CRC diagnosis (n = 3)

Included (n = 26,729)

**Population**

**Exclusion**

**Inclusion**

**Supplementary Figure 2**. Standardised incidence ratio of multiple primary cancers by sex in individuals diagnosed with index colorectal cancer


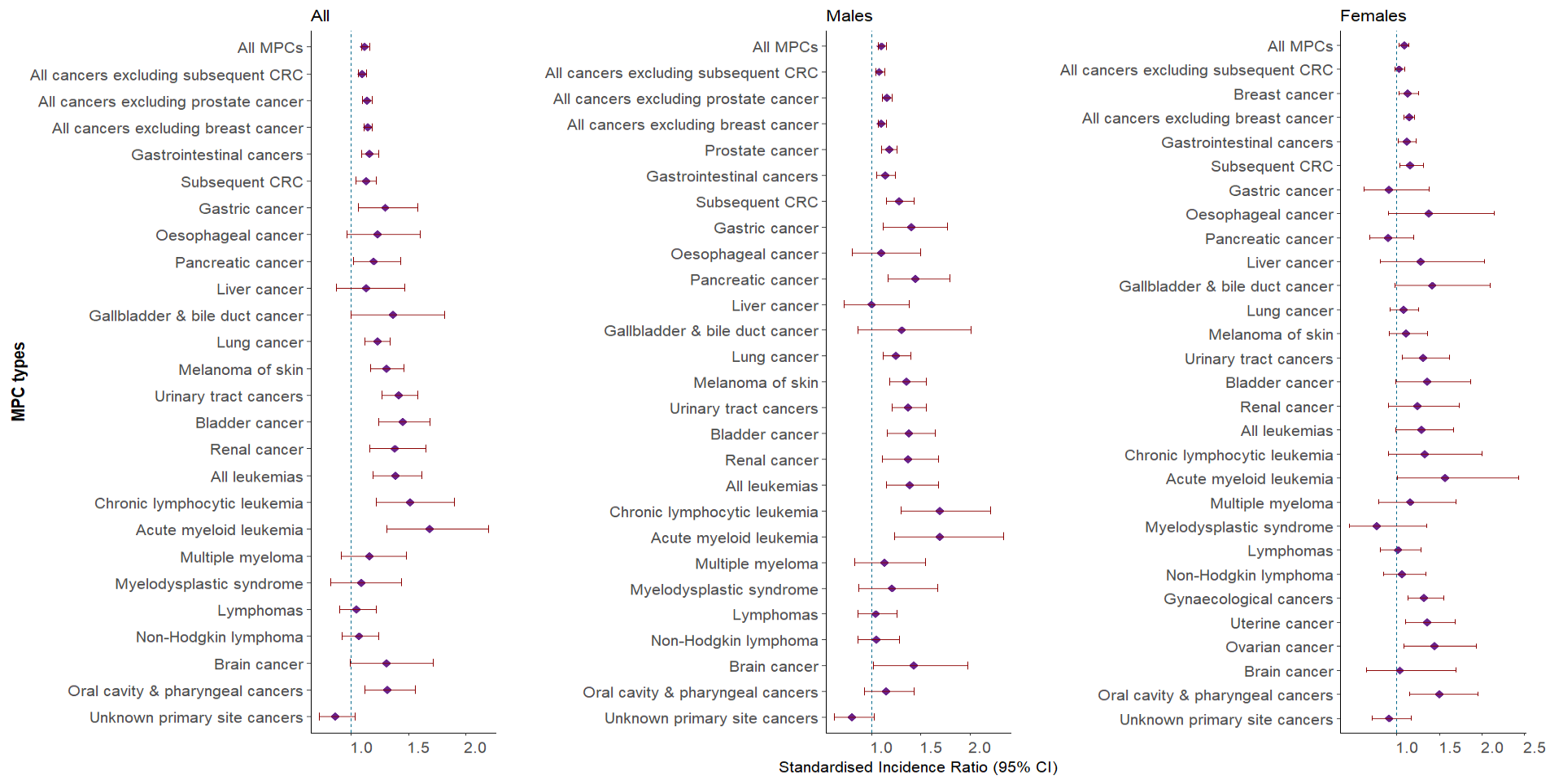


The purple diamond shapes represent the point estimates of the standardised incidence ratios (SIRs) for the respective MPC types; The horizontal lines crossing the diamonds indicate the 95% confidence intervals; the vertical dashed blue line marks the reference SIR value of 1, indicating neither increased nor decreased risk.

**Supplementary Figure 3**: Trends of multiple primary cancers in individuals diagnosed with colorectal cancer in South Australia between 1990 and 2017 by location of index colorectal cancer

Panel **A** presented APC of MPC trend in both sexes combined among individuals diagnosed with index colon cancer, including all site cancers; panel **B** presented APC of MPC trend in both sexes combined among individuals diagnosed with index rectal cancer, including all site cancers

1. **SUPPLEMENTARY DISCUSSION**

**3.1. Risk of MPC for selected cancer types**

Multiple primary cancers pose a growing challenge for cancer survivors by increasing treatment costs, reducing the quality of life, affecting mental well-being, substantially increasing the risk of suicide, and reducing survival rates.^23-26^ In this study, prostate cancer, subsequent CRC, lung cancer, breast cancer, leukaemia, lymphoma, urinary tract cancers, and skin melanoma were the most prevalent cancers, collectively constituting over two-thirds of the MPCs in CRC survivors. CRC survivors had a 12% higher risk of developing subsequent MPC compared with the expected risk in the general population. The risk of developing subsequent cancer is significantly higher for the prostate, colorectum, urinary tract, lung, gastric, small intestine, pancreatic, and blood cancers. The incidence of MPC had also increased significantly, with a 1.95% annual change from 1990 to 2017. Many studies report the risk of subsequent cancer in CRC survivors;^2, 5, 6, 27-29^ however, to our knowledge, this is the first study to comprehensively assess the risk of subsequent cancers among CRC survivors in Australia.

In this study, the incidence of lung cancers was also significantly higher among CRC survivors compared to the general population. Smoking is a well-known risk factor for many cancers, including lung cancer and CRC. According to the Australian Institute of Health and welfare (AIHW), 24.3%, 12.2% and 8.3% of Australians aged 14 years and older were daily smoker in 1991, 2016 and 2023, respectively, and nearly 30% of smokers were not planning to quit.^30^ Cancer Council Australia also reported that approximately 20% of annual cancer cases in Australia can be attributed to smoking.^31^ Romaszko-Wojtowicz et al., 2018 indicated that cancer survivors who continue smoking or quit only after their initial cancer diagnosis are at a significantly higher risk of developing a subsequent primary cancer earlier than non-smokers.^32^ A study of cancer survivors in Australia revealed that 63% of those who smoked at the time of their cancer diagnosis continued smoking afterward.^33^ This continued smoking has been associated with poor treatment outcomes, reduced life expectancy, and an increased likelihood of developing secondary cancers, including lung cancer and CRC.^32, 34^

In our study, CRC survivors were found to have a significantly elevated risk of melanoma compared to the general population. Previous research has reported conflicting findings regarding this association.^5, 28, 35, 36^ However, a recent systematic review of pooled results^37^ and studies conducted in Australia^5, 35^ have indicated an increased risk of melanoma among CRC survivors. This heightened risk may be partly attributable to geographic factors, as Australia has the highest incidence rate of malignant melanoma globally.^38^ In addition, some genetic mutations associated with CRC may also increase the risk of malignant melanoma. For instance, BRAF gene mutations, which are present in 10-20 % of CRC patients^39-41^, are also found in nearly half of melanoma cases.^39^

In this study, the risk of haematological malignancies was also significantly elevated in CRC survivors, but evidence in the literature is limited and inconsistent. Studies reported there is no significant increase in risk in CRC survivors,^5, 29^ while another reported a higher incidence of leukaemias among CRC survivors compared to the general population.^42^ One study even indicated a low risk of lymphatic and haematopoietic malignancy in colon cancer survivors.^2^ Conversely, a study by Lee et al. (2015) found that colon cancer survivors had a higher risk for haematological malignancies, but this risk was not observed in rectal cancer survivors.^29^ However, several reclassifications and changes in the histological codes for haematological malignancies over the past few decades have resulted in inconsistencies between the studies referenced. ^43, 44^ Furthermore, increased use of neoadjuvant and adjuvant chemotherapy for the treatment of solid cancers has been shown to be associated with therapy-related complications, including leukaemogenesis.^45-47^ Neo-adjuvant external beam radiation for the management of rectal cancer has also been shown to affect the hematopoietic system in the pelvic region, meaning haematological malignancies are long-term sequelae of therapy.^48, 49^ A study from the Surveillance Epidemiology and End Result (SEER) program showed that the risk of therapy-related acute myeloid leukaemia and myelodysplastic syndrome was significantly higher in patients treated with chemotherapy for 22 of 23 solid cancers, including rectal/rectosigmoid, but not colon cancer.^45^ This observation supports our finding that there is an increased risk of leukaemias among rectal cancer survivors. Compared to the general population, the risk of leukaemia is 45% in males, 67% in females, and 61% in all rectal cancer survivors (Supplementary Table 3).

- 1. **Risk of MPC by age, sex and location of index cancer**

The data from the current study suggest that the risk of developing MPCs is higher in both men and women. However, in absolute terms, males exhibit a two-fold higher risk compared to females, with an AER of 23.6 and 12.4 cases per 10,000 population for men and women, respectively. This disparity is likely to be a reflection of the higher incidence of cancer in males compared to females in the general population as gender differences in cancer susceptibility have been documented in epidemiological studies;^50, 51^ for most non-gender-specific anatomical sites, males have a higher risk of cancer than females.^50^ It has been hypothesised that the higher risk of cancer among men could be due to several physiological, genetic, epigenetic, and immunologic factors, including differences in the levels of testosterone and progesterone.^51, 52^ Higher testosterone levels in males have been implicated in promoting cell growth and tumorigenesis, even after adjusting for biological and environmental factors.^53, 54^ In contrast, the higher levels of progesterone and estrogen in women are associated with a lower risk for some non-sex-specific cancers, such as colon and pancreatic cancers.^52, 55, 56^ Moreover, behavioural and environmental factors that increase cancer risk tend to be more prevalent among men. Studies have shown that modifiable risk factors such as smoking, alcohol consumption, high body mass index and obesity, and dietary risks are more prevalent in men than in women.^57, 58^

The risk of MPCs increased regardless of the age at diagnosis of the index CRC. However, in CRC survivors who were <50 years old at the time of index CRC diagnosis, the risk of an MPC increased by 52% (95% CI: 32, 75) compared to 11% (95%CI: 7, 15) in patients who were >50 years old. These findings are consistent with previous studies, which also reported a high risk of MPCs in all age groups but a more pronounced risk in early-onset CRC survivors.^6, 28, 59^ The risk of MPCs in > 50-year-old survivors is likely associated with the overall age-related cancer risk.^60^ Several factors may explain why younger CRC survivors are at a greater risk of developing subsequent cancers. Firstly, there is a known association between early onset CRC and hereditary predisposition and family history.^61, 62^ Secondly, young-onset CRC patients are typically diagnosed with more advanced stage and poorly differentiated disease ^63, 64^ and are more likely to have advanced histological features, such as high tumour grade and mucinous or signet ring histology.^65, 66^ Thirdly, early-onset CRC is associated with distinctive molecular features, including epigenetic changes suggestive of global DNA hypomethylation, and a high proportion of these patients have a disease classified as molecular -1 subtype, which is characterised by a high degree of microsatellite instability (MSI), chronic inflammation, and expression of immunogenic markers.^67^ Dysfunctional DNA repair, immune regulation and apoptosis are involved in most forms of cancer and, therefore, may confer an increased risk for the development of MPCs. Fourthly, recent studies have also suggested that early-onset CRC patients have shown different gut microbiota profiles and host-microbiome associations compared to late-onset CRC patients for cancer-promoting and preventive microbial species.^68, 69^ Adnan et al. (2024) reported stronger microbial-host pathway interactions in early-onset CRC patients, particularly in pathways linked to CRC tumorigenesis, such as sulfur metabolism.^68^ Disruption in sulfur metabolism increases hydrogen sulfide concentrations, contributing to DNA damage, mucosal disruption, chronic inflammation, and carcinogenesis. In addition, the authors reported that early-onset CRC patients showed stronger microbial associations with DNA repair pathways, potentially increasing DNA instability and promoting carcinogenesis. Dysfunctional DNA repair, immune regulation and apoptosis are involved in most forms of cancer and, therefore, may confer an increased risk for the development of MPCs.^70-73^

Previous studies have shown that survivors with right-sided colon cancer have a higher risk of developing an MPC compared to left-sided colon and rectum.^1, 6, 16^ Our study also showed that right-sided colon cancer showed an increased risk of MPC in both sexes whereas left-sided colon cancer only increased the risk of MPC in males. Right-sided or proximally located colon tumours have distinctive clinicopathological, immunological and genetic features and are usually larger in size, higher T-stage and grade and more frequently of the mucinous histologic subtype compared to the distal colon and rectum.^74^ Furthermore, right-sided colon tumours often have a higher frequency of *BRAF* mutations, are of the MSI-high phenotype and express tumour-associated genetic markers (*CD44v6*, *CDX2,* *CD44*, *TOPK*, nuclear *β-catenin*, *BCL2*, *pERK*, *APAF-1*, *E-cadherin* and *p21*) and tumour-associated immune markers (CD68, CD163, FoxP3 and TIA-1).^74^ Moreover, proximal colon cancers are more likely to have a CpG island methylator phenotype and harbour *KRAS* mutations than distal colon and rectal tumours.^75^ The clinicopathological, immunological, and genetic profiles associated with proximal colon cancer may promote disease progression, which could limit treatment outcomes and increase the risk of subsequent primary cancer.

1. **REFERENCES**

1. Raj KP, Taylor TH, Wray C, Stamos MJ, Zell JA. Risk of second primary colorectal cancer among colorectal cancer cases: a population-based analysis. J Carcinog 2011;10:6.

2. Guan X, Jin Y, Chen Y, Jiang Z, Liu Z, Zhao Z, Yan P, Wang G, Wang X. The Incidence Characteristics of Second Primary Malignancy after Diagnosis of Primary Colon and Rectal Cancer: A Population Based Study. PLoS One 2015;10:e0143067.

3. Sung H, Hyun N, Leach CR, Yabroff KR, Jemal A. Association of First Primary Cancer With Risk of Subsequent Primary Cancer Among Survivors of Adult-Onset Cancers in the United States. Jama 2020;324:2521-35.

4. Pedersen JK, Engholm G, Skytthe A, Christensen K. Cancer and aging: Epidemiology and methodological challenges. Acta Oncol 2016;55 Suppl 1:7-12.

5. Dasgupta P, Youlden DR, Baade PD. Multiple primary cancers among colorectal cancer survivors in Queensland, Australia, 1996-2007. Cancer Causes Control 2012;23:1387-98.

6. Liang LA, Tseng YJ, Tanaka LF, Klug SJ. Second primary cancer among 217702 colorectal cancer survivors: An analysis of national German cancer registry data. Int J Cancer 2023;153:1459-71.

7. Working Group Report. International rules for multiple primary cancers (ICD-0 third edition). Eur J Cancer Prev 2005;14:307-8.

8. International Agency for Research on Cancer. International Classification of Diseases for Oncology (ICD-O). 3rd ed. Geneva: World Health Organization; 2013 [cited 2025 Mar 20]. Available from: <https://iris.who.int/bitstream/handle/10665/96612/9789241548496_eng.pdf?sequence=1>.

9. Zhang B, Guo K, Zheng X, Sun L, Shen M, Ruan S. Risk of Second Primary Malignancies in Colon Cancer Patients Treated With Colectomy. Front Oncol 2020;10:1154.

10. Jia H, Li Q, Yuan J, Sun X, Wu Z. Second Primary Malignancies in Patients with Colorectal Cancer: A Population-Based Analysis. Oncologist 2020;25:e644-e50.

11. Cancer in South Australia 2020 – with projections to 2023. Adelaide: South Australian Cancer Registry, Prevention and Population Health Directorate, Wellbeing SA, Government of South Australia, 2022.

12. International rules for multiple primary cancers (ICD-0 third edition). Eur J Cancer Prev 2005;14:307-8.

13. Becher H, Winkler V. Estimating the standardized incidence ratio (SIR) with incomplete follow-up data. BMC Med Res Methodol 2017;17:55.

14. Naing NN. Easy way to learn standardization: direct and indirect methods. Malays J Med Sci 2000;7:10-5.

15. Registry. SAC. Cancer Incidence and Mortality: All Cancers, South Australia Cancer Registry, 2001-2020; Public Users - Site Specific Data, Cancer Registry, Government of South Australia. Available at: <https://prod-apsoutheast-a.online.tableau.com/#/site/wellbeingsaopendataportal/views/PublicUsers-SiteSpecificData_16850847796760/PublicUsers-SiteSpecificData?:iid=4>, Accessed date: April 2023.

16. Liu L, Lemmens VE, De Hingh IH, de Vries E, Roukema JA, van Leerdam ME, Coebergh JW, Soerjomataram I. Second primary cancers in subsites of colon and rectum in patients with previous colorectal cancer. Dis Colon Rectum 2013;56:158-68.

17. Odani S, Nakata K, Inoue M, Kato M, Saito MK, Morishima T, Hashii Y, Hara J, Kawa K, Miyashiro I. Incidence of second primary cancers among survivors of childhood cancer: A population-based study, Osaka, Japan, 1975-2015. Cancer Sci 2023;114:1142-53.

18. AIHW. Cancer rankings data visualisation: Cancer data in Australia. Available at: <https://www.aihw.gov.au/reports/cancer/cancer-data-in-australia/contents/rankings>; accessed date: 3 July 2024

19. Ahmed F, Goodman MT, Kosary C, Ruiz B, Wu XC, Chen VW, Correa CN. Excess risk of subsequent primary cancers among colorectal carcinoma survivors, 1975-2001. Cancer 2006;107:1162-71.

20. Weir HK, Johnson CJ, Ward KC, Coleman MP. The effect of multiple primary rules on cancer incidence rates and trends. Cancer Causes Control 2016;27:377-90.

21. National Cancer Institute. Joinpoint Trend Analysis Software.Division of Cancer Control and Population Sciences, National Cancer Institute. Version 5.1.0 - April 9, 2024. available at: <https://surveillance.cancer.gov/joinpoint/> 2024.

22. Ilic L, Haidinger G, Simon J, Hackl M, Schernhammer E, Papantoniou K. Trends in female breast cancer incidence, mortality, and survival in Austria, with focus on age, stage, and birth cohorts (1983-2017). Sci Rep 2022;12:7048.

23. Yang H, Qu Y, Shang Y, Wang C, Wang J, Lu D, Song H. Increased Risk of Suicide among Cancer Survivors Who Developed a Second Malignant Neoplasm. Comput Intell Neurosci 2022;2022:2066133.

24. Gotay CC, Ransom S, Pagano IS. Quality of life in survivors of multiple primary cancers compared with cancer survivor controls. Cancer 2007;110:2101-9.

25. Gordon LG, Wood C, Tothill RW, Webb PM, Schofield P, Mileshkin L. Healthcare Costs Before and After Diagnosis of Cancer of Unknown Primary Versus Ovarian Cancer in Australia. Pharmacoecon Open 2023;7:111-20.

26. Park HY, Yang HJ, Jeon SM, Lee IH, Kwon JW. Survival and medical costs of melanoma patients with subsequent cancer diagnoses: A South Korean population-based retrospective cohort study. Asia Pac J Clin Oncol 2022;18:e211-e19.

27. Collatuzzo G, Ferrante M, Ippolito A, Di Prima A, Colarossi C, Scarpulla S, Boffetta P, Sciacca S. Second Primary Cancers following Colorectal Cancer in Sicily, Italy. Cancers (Basel) 2022;14.

28. He X, Wu W, Ding Y, Li Y, Si J, Sun L. Excessive risk of second primary cancers in young-onset colorectal cancer survivors. Cancer Med 2018;7:1201-10.

29. Lee YT, Liu CJ, Hu YW, Teng CJ, Tzeng CH, Yeh CM, Chen TJ, Lin JK, Lin CC, Lan YT, Wang HS, Yang SH, et al. Incidence of Second Primary Malignancies Following Colorectal Cancer: A Distinct Pattern of Occurrence Between Colon and Rectal Cancers and Association of Co-Morbidity with Second Primary Malignancies in a Population-Based Cohort of 98,876 Patients in Taiwan. Medicine (Baltimore) 2015;94:e1079.

30. AIHW, Tobacco smoking in the NDSHS.Available at: <https://www.aihw.gov.au/reports/smoking/tobacco-smoking-ndshs>, Accessed on: 3 July 2024.

31. Australia CC. Smoking: Reduce the harms caused by smoking. Aviable at <https://www.cancer.org.au/cancer-information/causes-and-prevention/smoking>, accsed on: 3 July 2024.

32. Romaszko-Wojtowicz A, Buciński A, Doboszyńska A. Impact of smoking on multiple primary cancers survival: a retrospective analysis. Clin Exp Med 2018;18:391-97.

33. Paul CL, Tzelepis F, Boyes AW, D'Este C, Sherwood E, Girgis A. Continued smoking after a cancer diagnosis: a longitudinal study of intentions and attempts to quit. J Cancer Surviv 2019;13:687-94.

34. Romaszko-Wojtowicz A, Lorenc A, Buciński A, Doboszyńska A. Effects of Tobacco Smoking on the Survivability of Patients with Multiple Cancers and Single Lung Cancer. Int J Environ Res Public Health 2022;19.

35. Youlden DR, Baade PD. The relative risk of second primary cancers in Queensland, Australia: a retrospective cohort study. BMC Cancer 2011;11:83.

36. Phipps AI, Chan AT, Ogino S. Anatomic subsite of primary colorectal cancer and subsequent risk and distribution of second cancers. Cancer 2013;119:3140-7.

37. Robertson D, Ng SK, Baade PD, Lam AK. Risk of extracolonic second primary cancers following a primary colorectal cancer: a systematic review and meta-analysis. Int J Colorectal Dis 2022;37:541-51.

38. De Pinto G, Mignozzi S, La Vecchia C, Levi F, Negri E, Santucci C. Global trends in cutaneous malignant melanoma incidence and mortality. Melanoma Res 2024;34:265-75.

39. Safaee Ardekani G, Jafarnejad SM, Tan L, Saeedi A, Li G. The prognostic value of BRAF mutation in colorectal cancer and melanoma: a systematic review and meta-analysis. PLoS One 2012;7:e47054.

40. Phipps AI, Buchanan DD, Makar KW, Burnett-Hartman AN, Coghill AE, Passarelli MN, Baron JA, Ahnen DJ, Win AK, Potter JD, Newcomb PA. BRAF mutation status and survival after colorectal cancer diagnosis according to patient and tumor characteristics. Cancer Epidemiol Biomarkers Prev 2012;21:1792-8.

41. Hughes LA, Williamson EJ, van Engeland M, Jenkins MA, Giles GG, Hopper JL, Southey MC, Young JP, Buchanan DD, Walsh MD, van den Brandt PA, Alexandra Goldbohm R, et al. Body size and risk for colorectal cancers showing BRAF mutations or microsatellite instability: a pooled analysis. Int J Epidemiol 2012;41:1060-72.

42. Yang J, Wu F, An H, Gan H. Incidence and risk outcomes of second primary malignancy of patients with post-operative colorectal cancer. Int J Colorectal Dis 2023;38:88.

43. Zhang N, Wu J, Wang Q, Liang Y, Li X, Chen G, Ma L, Liu X, Zhou F. Global burden of hematologic malignancies and evolution patterns over the past 30 years. Blood Cancer J 2023;13:82.

44. Alaggio R, Amador C, Anagnostopoulos I, Attygalle AD, Araujo IBO, Berti E, Bhagat G, Borges AM, Boyer D, Calaminici M, Chadburn A, Chan JKC, et al. The 5th edition of the World Health Organization Classification of Haematolymphoid Tumours: Lymphoid Neoplasms. Leukemia 2022;36:1720-48.

45. Morton LM, Dores GM, Schonfeld SJ, Linet MS, Sigel BS, Lam CJK, Tucker MA, Curtis RE. Association of Chemotherapy for Solid Tumors With Development of Therapy-Related Myelodysplastic Syndrome or Acute Myeloid Leukemia in the Modern Era. JAMA Oncol 2019;5:318-25.

46. Lehrer S, Rheinstein PH. Increased Risk of Acute Myelogenous Leukemia After Early Onset but Not Late-Onset Colorectal Cancer. Am J Clin Oncol 2020;43:263-69.

47. Vakili-Sadeghi M, Omranpour M. Chronic myeloid leukemia following colon cancer treatment: A case report and literature review. Caspian J Intern Med 2013;4:739-42.

48. Stein EM, Pareek V, Kudlowitz D, Douer D, Tallman MS. Acute leukemias following a diagnosis of colorectal cancer: Are they therapy-related? Blood 2012;120:1453.

49. Wang TH, Liu CJ, Chao TF, Chen TJ, Hu YW. Second primary malignancy risk after radiotherapy in rectal cancer survivors. World J Gastroenterol 2018;24:4586-95.

50. Jackson SS, Marks MA, Katki HA, Cook MB, Hyun N, Freedman ND, Kahle LL, Castle PE, Graubard BI, Chaturvedi AK. Sex disparities in the incidence of 21 cancer types: Quantification of the contribution of risk factors. Cancer 2022;128:3531-40.

51. Dorak MT, Karpuzoglu E. Gender differences in cancer susceptibility: an inadequately addressed issue. Front Genet 2012;3:268.

52. Zhang YL, Wen XD, Guo X, Huang SQ, Wang TT, Zhou PT, Li W, Zhou LF, Hu YH. Progesterone suppresses the progression of colonic carcinoma by increasing the activity of the GADD45α/JNK/c‑Jun signalling pathway. Oncol Rep 2021;45.

53. Hyde Z, Flicker L, McCaul KA, Almeida OP, Hankey GJ, Chubb SA, Yeap BB. Associations between testosterone levels and incident prostate, lung, and colorectal cancer. A population-based study. Cancer Epidemiol Biomarkers Prev 2012;21:1319-29.

54. Watts EL, Perez-Cornago A, Knuppel A, Tsilidis KK, Key TJ, Travis RC. Prospective analyses of testosterone and sex hormone-binding globulin with the risk of 19 types of cancer in men and postmenopausal women in UK Biobank. Int J Cancer 2021;149:573-84.

55. Goncharov AI, Maslakova AA, Polikarpova AV, Bulanova EA, Guseva AA, Morozov IA, Rubtsov PM, Smirnova OV, Shchelkunova TA. Progesterone inhibits proliferation and modulates expression of proliferation-Related genes in classical progesterone receptor-negative human BxPC3 pancreatic adenocarcinoma cells. J Steroid Biochem Mol Biol 2017;165:293-304.

56. Gizard F, Robillard R, Gervois P, Faucompré A, Révillion F, Peyrat JP, Hum WD, Staels B. Progesterone inhibits human breast cancer cell growth through transcriptional upregulation of the cyclin-dependent kinase inhibitor p27Kip1 gene. FEBS Lett 2005;579:5535-41.

57. Brown KF, Rumgay H, Dunlop C, Ryan M, Quartly F, Cox A, Deas A, Elliss-Brookes L, Gavin A, Hounsome L, Huws D, Ormiston-Smith N, et al. The fraction of cancer attributable to modifiable risk factors in England, Wales, Scotland, Northern Ireland, and the United Kingdom in 2015. Br J Cancer 2018;118:1130-41.

58. Chen W, Xia C, Zheng R, Zhou M, Lin C, Zeng H, Zhang S, Wang L, Yang Z, Sun K, Li H, Brown MD, et al. Disparities by province, age, and sex in site-specific cancer burden attributable to 23 potentially modifiable risk factors in China: a comparative risk assessment. Lancet Glob Health 2019;7:e257-e69.

59. Liang YH, Shao YY, Chen HM, Lai CL, Lin ZZ, Kuo RN, Cheng AL, Yeh KH, Lai MS. Young patients with colorectal cancer have increased risk of second primary cancers. Jpn J Clin Oncol 2015;45:1029-35.

60. NCI. National cancer institute: Age and Cancer Risk; available at: <https://www.cancer.gov/about-cancer/causes-prevention/risk/age>; date acessed: 9 July 2024.

61. You YN, Moskowitz JB, Chang GJ, Mork ME, Rodriguez-Bigas MA, Bednarski BK, Messick CA, Tillman MM, Skibber JM, Nguyen ST, Kopetz S, Vilar E. Germline Cancer Risk Profiles of Patients With Young-Onset Colorectal Cancer: Findings From a Prospective Universal Germline Testing and Telegenetics Program. Dis Colon Rectum 2023;66:531-42.

62. Ochs-Balcom HM, Kanth P, Cannon-Albright LA. Early-onset colorectal cancer risk extends to second- and third-degree relatives. Cancer Epidemiol 2021;73:101973.

63. Boardman LA, Johnson RA, Petersen GM, Oberg AL, Kabat BF, Slusser JP, Wang L, Morlan BW, French AJ, Smyrk TC, Lindor NM, Thibodeau SN. Higher frequency of diploidy in young-onset microsatellite-stable colorectal cancer. Clin Cancer Res 2007;13:2323-8.

64. Ahnen DJ, Wade SW, Jones WF, Sifri R, Mendoza Silveiras J, Greenamyer J, Guiffre S, Axilbund J, Spiegel A, You YN. The increasing incidence of young-onset colorectal cancer: a call to action. Mayo Clin Proc 2014;89:216-24.

65. Lawler T, Parlato L, Warren Andersen S. The histological and molecular characteristics of early-onset colorectal cancer: a systematic review and meta-analysis. Front Oncol 2024;14:1349572.

66. You YN, Xing Y, Feig BW, Chang GJ, Cormier JN. Young-onset colorectal cancer: is it time to pay attention? Arch Intern Med 2012;172:287-9.

67. Antelo M, Balaguer F, Shia J, Shen Y, Hur K, Moreira L, Cuatrecasas M, Bujanda L, Giraldez MD, Takahashi M, Cabanne A, Barugel ME, et al. A high degree of LINE-1 hypomethylation is a unique feature of early-onset colorectal cancer. PLoS One 2012;7:e45357.

68. Adnan D, Trinh JQ, Sharma D, Alsayid M, Bishehsari F. Early-onset Colon Cancer Shows a Distinct Intestinal Microbiome and a Host-Microbe Interaction. Cancer Prev Res (Phila) 2024;17:29-38.

69. Xiong H, Wang J, Chang Z, Hu H, Yuan Z, Zhu Y, Hu Z, Wang C, Liu Y, Wang Y, Wang G, Tang Q. Gut microbiota display alternative profiles in patients with early-onset colorectal cancer. Front Cell Infect Microbiol 2022;12:1036946.

70. Huang H, Liu Y, Wen Z, Chen C, Wang C, Li H, Yang X. Gut microbiota in patients with prostate cancer: a systematic review and meta-analysis. BMC Cancer 2024;24:261.

71. Wei YF, Huang MS, Huang CH, Yeh YT, Hung CH. Impact of Gut Dysbiosis on the Risk of Non-Small-Cell Lung Cancer. Int J Environ Res Public Health 2022;19.

72. Wei Z, Yang B, Tang T, Xiao Z, Ye F, Li X, Wu S, Huang JG, Jiang S. Gut microbiota and risk of five common cancers: A univariable and multivariable Mendelian randomization study. Cancer Med 2023;12:10393-405.

73. Chen G, Kuang Z, Li F, Li J. The causal relationship between gut microbiota and leukemia: a two-sample Mendelian randomization study. Front Microbiol 2023;14:1293333.

74. Minoo P, Zlobec I, Peterson M, Terracciano L, Lugli A. Characterization of rectal, proximal and distal colon cancers based on clinicopathological, molecular and protein profiles. Int J Oncol 2010;37:707-18.

75. Slattery ML, Curtin K, Wolff RK, Boucher KM, Sweeney C, Edwards S, Caan BJ, Samowitz W. A comparison of colon and rectal somatic DNA alterations. Dis Colon Rectum 2009;52:1304-11.
